# A global assembly of adult female mosquito mark-release-recapture data to inform the control of mosquito-borne pathogens

**DOI:** 10.1186/1756-3305-7-276

**Published:** 2014-06-19

**Authors:** Carlos A Guerra, Robert C Reiner, T Alex Perkins, Steve W Lindsay, Janet T Midega, Oliver J Brady, Christopher M Barker, William K Reisen, Laura C Harrington, Willem Takken, Uriel Kitron, Alun L Lloyd, Simon I Hay, Thomas W Scott, David L Smith

**Affiliations:** 1 Fogarty International Center, National Institutes of Health, Bethesda, MD, USA; 2 Center for Disease Dynamics, Economics & Policy, Washington, DC, USA; 3 Department of Entomology, University of California, Davis, CA, USA; 4 Department of Disease Control, London School of Hygiene and Tropical Medicine, London, UK; 5 School of Biological and Biomedical Sciences, Durham University, Durham, UK; 6 Pathogen, Vector and Human Biology Unit, KEMRI–University of Oxford–Wellcome Trust Research Programme, Kilifi, Kenya; 7 Department of Life Sciences, Imperial College London, London, UK; 8 Spatial Ecology and Epidemiology Group, Department of Zoology, Tinbergen Building, University of Oxford, South Parks Road, Oxford, United Kingdom; 9 Center for Vectorborne Diseases, Department of Pathology, Microbiology, and Immunology, School of Veterinary Medicine, University of California, Davis, CA, USA; 10 Department of Entomology, Cornell University, Ithaca, NY, USA; 11 Laboratory of Entomology, Wageningen University, Wageningen, The Netherlands; 12 Department of Environmental Sciences, Emory University, Atlanta, GA, USA; 13 Department of Mathematics and Biomathematics Graduate Program, North Carolina State University, Raleigh, NC, USA; 14 Department of Epidemiology, Bloomberg School of Public Health, Johns Hopkins University, Baltimore, MD, USA

**Keywords:** Mosquito, Vector, Mark-release-recapture, Database, Pathogen transmission models

## Abstract

**Background:**

Pathogen transmission by mosquitos is known to be highly sensitive to mosquito bionomic parameters. Mosquito mark-release-recapture (MMRR) experiments are a standard method for estimating such parameters including dispersal, population size and density, survival, blood feeding frequency and blood meal host preferences.

**Methods:**

We assembled a comprehensive database describing adult female MMRR experiments. Bibliographic searches were used to build a digital library of MMRR studies and selected data describing the reported outcomes were extracted.

**Results:**

The resulting database contained 774 unique adult female MMRR experiments involving 58 vector mosquito species from the three main genera of importance to human health: *Aedes*, *Anopheles* and *Culex*. Crude examination of these data revealed patterns associated with geography as well as mosquito genus, consistent with bionomics varying by species-specific life history and ecological context. Recapture success varied considerably and was significantly different amongst genera, with 8, 4 and 1% of adult females recaptured for *Aedes*, *Anopheles* and *Culex* species, respectively. A large proportion of experiments (59%) investigated dispersal and survival and many allowed disaggregation of the release and recapture data. Geographic coverage was limited to just 143 localities around the world.

**Conclusions:**

This MMRR database is a substantial contribution to the compilation of global data that can be used to better inform basic research and public health interventions, to identify and fill knowledge gaps and to enrich theory and evidence-based ecological and epidemiological studies of mosquito vectors, pathogen transmission and disease prevention. The database revealed limited geographic coverage and a relative scarcity of information for vector species of substantial public health relevance. It represents, however, a wealth of entomological information not previously compiled and of particular interest for mosquito-borne pathogen transmission models.

## Background

Mosquitoes are important vectors of viruses and parasites of human, veterinary and wildlife health concern [1]. Major mosquito-borne infectious diseases of humans include malaria, dengue, filariasis, chikungunya fever, Rift Valley fever and the arboviral encephalitides, including Japanese encephalitis and West Nile neuroinvasive viral disease. Infections can cause high morbidity and mortality and are of great public health concern in endemic areas. Globally, malaria parallels HIV/AIDS and is slightly behind diarrhoeal disease in terms of disability-adjusted life years lost [2]. The current estimated global burden of dengue is higher (390 million infections per year) than previously appreciated [3] and will likely increase as the range of *Aedes* expands [4,5] and as humans are increasingly concentrated in high risk urban areas [6]. Lymphatic filariasis often leads to permanent disability and its global burden ranks high amongst other neglected tropical diseases [2]. West Nile virus is the most widespread mosquito-borne neuropathogen and has dramatically expanded its geographic range across the Western Hemisphere after its introduction into North America in 1999 [7].

The vectorial capacity [8] is the ideal theoretical metric for describing the potential intensity of mosquito-borne pathogen transmission and responses to control. There is a substantial body of research, including studies utilizing mosquito mark-release-recapture (MMRR) as a method, which have measured the intensity of transmission and other mosquito bionomic parameters that affect pathogen transmission [9,10]. Vectorial capacity contains the entomological components of a pathogen’s basic reproductive number (*R*_0_) and offers clarification about the relative importance of different aspects of mosquito behavior and ecology for transmission [11], i.e., mosquito population density relative to the pathogen’s host population density, daily mosquito survival, the blood index describing the proportion of bites taken from a pathogen’s host, the blood feeding rate describing the duration of the gonotrophic cycle or the interval between two successive blood meals, and the duration of the extrinsic incubation period. Vectorial capacity posits that transmission will be reduced linearly by reductions in mosquito density, quadratically by slowing the rate at which mosquitoes bite amplifying hosts, and approximately cubically with increases in mosquito mortality [12]. Recent theoretical developments refined the connection between mosquito ecology and transmission further [13,14] by, for example, identifying that the scale of mosquito movement determines the spatial scale of transmission and thereby the number of hosts that can potentially become infected by a single mosquito [15]. Studies of transmission have focused on human biting rates and the entomological inoculation rates, depending on the pathogen [16], but there are persistent questions about the precision and accuracy of such estimates [10]. MMRR provide a method for cross-validation and unique insight into how mosquito movement patterns result in their dispersal through the environment [17]. Quantitative estimates of these parameters have been used to inform models of malaria [18] and of other mosquito-transmitted pathogens [9,19].

Although models have identified those aspects of mosquito ecology that are most important for transmission and that should be the most promising targets for control, field measurements of those parameters are required to inform model-based evaluations of different control measures in different ecological contexts [20,21]. An aspirational goal is to develop an evidence-base that could establish average bionomic parameters for various vector species and to explain and predict variation in mosquito bionomic parameters in various ecological settings across a species’ geographical range. These parameters would be useful for implementing control as well as for modeling the pathogens. An important common feature in the measurement of entomological parameters describing transmission is that historically they involved marking and releasing mosquitoes and recapturing them after some period of time. These MMRR experiments have been performed hundreds of times over decades on many different species, in a variety of locations and for many different purposes [22].

Despite their abundance and known importance for informing transmission models of mosquito-borne pathogens, data from the numerous MMRR studies have not been compiled into a single repository so that their usefulness in this context can be critically assessed. Global databases are increasingly assembled to support vector-borne disease entomological and epidemiological research [23,24]. A notable example is the recent effort in malaria research to provide data repositories on disease prevalence [24] and vector occurrence [25-27]. These large data sets have been used as the basis of geo-spatial models of disease endemicity [28-30] and of the distribution of mosquito vectors [31] at national, regional and global scales. They have also provided the scientific community with unique opportunities to explore intrinsic relationships between entomological and epidemiological variables and to advance the theory behind practical, disease prevention questions [32]. Here, we report on an extensive global database of MMRR studies conducted over the last century with the ultimate aim to help improve mosquito-borne pathogen transmission models. This paper is the first on this topic and as such is limited to presenting a description of the database and preliminary analyses that give a general overview of its contents, strengths and limitations to inform modeling approaches.

## Methods

### Assembling a bibliographic archive of MMRR studies

A digital library of MMRR field studies was assembled in a bibliographic manager. References were retrieved from searches in two digital bibliographic databases: PubMed (http://www.ncbi.nlm.nih.gov/pubmed) and Google Scholar (scholar.google.com). These were chosen because of their open access credentials and easy integration with digital libraries. The following keywords were used in different Boolean search combinations: mosquito, vector, mark, release, recapture and capture. No constraints were applied to date of publication and both published (e.g. journal articles, book chapters) and unpublished literature sources (e.g. postgraduate theses, reports) in any of four languages (English, Spanish, French and Portuguese) were considered. Candidate references were filtered manually after checking their titles and abstracts to narrow the list. Full-text copies were sought for those references classified as potential MMRR data sources for any of the three mosquito genera of greatest relevance for pathogen transmission to humans: *Aedes*, *Anopheles* and *Culex*[33]. MMRR studies on species belonging to other genera of medical importance (e.g. *Mansonia*, *Haemagogus*, *Sabethes*) were found to be too limited in number to provide meaningful insights into the influence of genus on the outcome of MMRR studies, and, hence, were not considered.

### Assembling MMRR data and ancillary information

A relational database was designed to store MMRR and ancillary data extracted from the selected literature. Data specifically related to the MMRR experiments included: marking and recapturing methods; numbers of mosquitoes released and recaptured; number, timing and location of releases and recaptures; and estimates of adult mosquito population size, daily survival probability, duration of the gonotrophic cycle, and distances travelled. An individual MMRR experiment, representing a data record, was defined as a single experimental release event conducted at a specific time and/or study site. Single records were those where uniquely marked study cohorts were established and/or different species were studied as part of the same study. In other words, an experiment releasing two distinctly marked cohorts produced two individual MMRR records in the database. The database was designed to keep data as disaggregated in time and space as possible; i.e., if MMRR experimental results were presented as releases and recaptures by day and/or distance, custom tables also captured this level of disaggregation.

Ancillary data included information about the study area such as geographic location, concurrent weather conditions (i.e., wind speed, temperature and humidity), land cover and human population. The database was linked to the digital library *via* unique record identifiers, so that all the MMRR data can be traced back to their original source. A list and description of the main database fields can be found in the Additional file 1.

### MMRR data analyses

Two filters were applied on the database prior to analyses. First, data were subset into *Aedes*, *Anopheles* and *Culex*. Then, and despite being included in the database for comprehensiveness, adult male mosquito MMRR data were excluded from the analyses. Although MMRR studies have been especially useful in estimating the mating competitiveness of sterile or genetically altered males [34], entomological parameters of mosquito-borne pathogen transmission concern exclusively blood-feeding, adult females.

The objectives of the MMRR data summaries and analyses presented here were to: (i) provide the reader with a general idea of the database contents, namely the number of references sourced, the number of MMRR data records they produced, the spatial and temporal distributions of these data, and the level of data disaggregation achieved in the database; (ii) explore the diversity of methodological approaches used at the various steps of the different experimental designs; and (iii) explore statistical differences in experimental outcomes, measured as the “recapture success”, and possible factors that account for these differences. Recapture success is defined herein as the ratio of the total number of marked adult female mosquitoes recaptured to the total number of marked female mosquitoes released in any given experiment [35], and is expressed hereafter as the “percent recaptured”.

## Results

### The MMRR digital library

The inclusive keyword-search combinations used in PubMed and Google Scholar produced an initial digital library containing 10,559 references. This list was narrowed to 528 candidate references possibly containing MMRR data for *Aedes*, *Anopheles* or *Culex* species according to their titles and abstracts and for which full-text copies were sought. The final digital library contained 212 relevant MMRR references, from which full-text versions for 194 were available for data extraction. In the end, 161 references contributed MMRR data (these were considered the original sources for the data) and 13 references contained data already reported by those considered original sources (i.e., multiple papers referring to the same MMRR data). Twenty sources for which full-text copies were obtained did not prove useful for data extraction because only limited information was available on the MMRR data described. The 161 original data sources included 158 published articles, one post-graduate thesis and two reports (Additional file 2). Table 1 shows that 47.2% of the consulted references concerned MMRR studies on *Anopheles* mosquitoes. Most studies (60%), regardless of species, measured adult female mosquito dispersal and/or survival (Table 2).

**Table 1:** Number of MMRR sources and corresponding records assembled by genus

**Genus**	**Sources**	**Total MMRR records**	**MMRR using adult females**
*Aedes*	48	306	287
*Anopheles*	76	298	291
*Culex*	37	196	196
**Total**	**161**	**800**	**774**

Records investigating adult female mosquitoes are listed and represent the sample sizes for further analyses.

**Table 2:** Frequency of MMRR studies by their main objectives

**Study objective**	**Any**
Dispersal	76
Survival	58
Gonotrophic cycle duration	26
Population size	25
Other	38

### The MMRR data

#### General summaries

The 161 original sources produced 800 individual MMRR experiments on 58 mosquito species within the three genera considered: 14 *Aedes*, 33 *Anopheles*, and 11 *Culex* species. Twenty-six records were excluded from the analyses because they reported only data from adult male mosquitoes. Although a few studies (n = 24) did not discriminate sex when reporting their results (i.e., numbers of males and females released and recaptured), these were kept for general summaries, but automatically excluded from recapture success analyses that required specific numbers of females released and recaptured. Amongst the 774 experiments considered, 37.1% concerned *Aedes*, 37.6% *Anopheles* and 25.3% *Culex* species (Table 1). The frequency of MMRR records by species is presented in Table 3, which shows that three species (*Aedes aegypti*, *Culex tarsalis* and *Anopheles culicifacies*) accounted for 43.2% of the MMRR data records. All of the 161 original sources are listed in Additional file 2, for reference. The 800 MMRR data records they produced are available in raw format in three tables contained within Additional file 3.

**Table 3:** MMRR records by mosquito species

**Mosquito species**	**MMRR records**	**MMRR data disaggregation**
*Aedes aegypti*	163	87
*Culex tarsalis*	113	43
*Anopheles culicifacies*	58	26
*Aedes cantans*	47	2
*Anopheles gambiae* s. l.	31	17
*Culex quinquefasciatus*	31	8
*Aedes albopictus*	25	22
*Anopheles vestitipennis*	19	9
*Anopheles darlingi*	17	12
*Anopheles stephensi*	16	0
*Aedes triseriatus*	16	3
*Anopheles funestus*	15	5
*Culex pipiens*	15	6
*Anopheles albimanus*	15	7
*Anopheles quadrimaculatus*	14	7
*Anopheles farauti*	11	9
*Anopheles subpictus*	11	1

The table also shows the number of MMRR records for which data disaggregation (in time and/or distance) was available. Only species for which ten or more MMRR experiments were recorded are shown.

### Spatial distribution of the MMRR data

**Figure 1:**
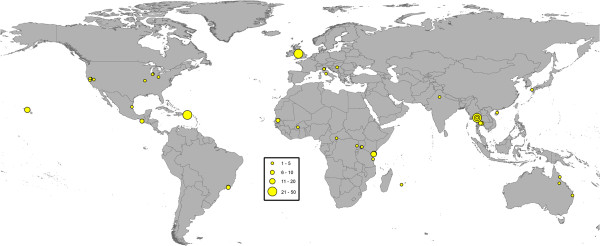
**Geographic distribution of MMRR study sites for *Aedes*.** Circle sizes represent the number of MMRR experiments conducted at each study site.

**Figure 2:**
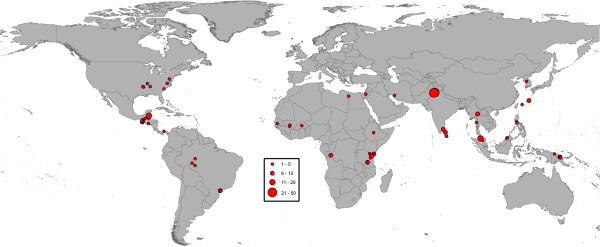
**Geographic distribution of MMRR study sites for *Anopheles*.** Circle sizes represent the number of MMRR experiments conducted at each study site.

**Figure 3:**
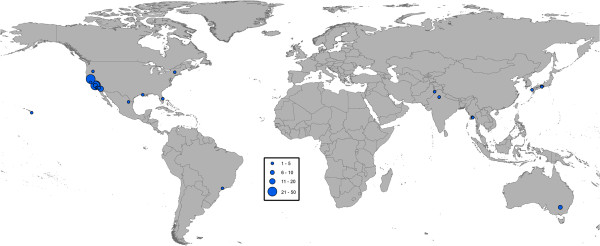
**Geographic distribution of MMRR study sites for *Culex*.** Circle sizes represent the number of MMRR experiments conducted at each study site.

The 774 MMRR experiments were conducted in 143 different study sites across 35 countries (Figures 1, 2 and 3). In general, the data revealed aggregation of study site geographic location with *Culex* data showing the most clustering and *Anopheles* the least. In the case of *Culex* and *Aedes* studies, 41.3% and 32.8% of the MMRR records, respectively, were reported in just three study sites. For *Culex*, these were all located in California, USA. For *Aedes*, one site was in the UK, one in Puerto Rico and one in Thailand. In the case of *Anopheles* studies, two study sites in Pakistan produced more than twenty MMRR records each, representing 21.3% of all the MMRR data assembled for anopheline mosquitoes.

#### ***Temporal distribution of the MMRR data***

**Figure 4:**
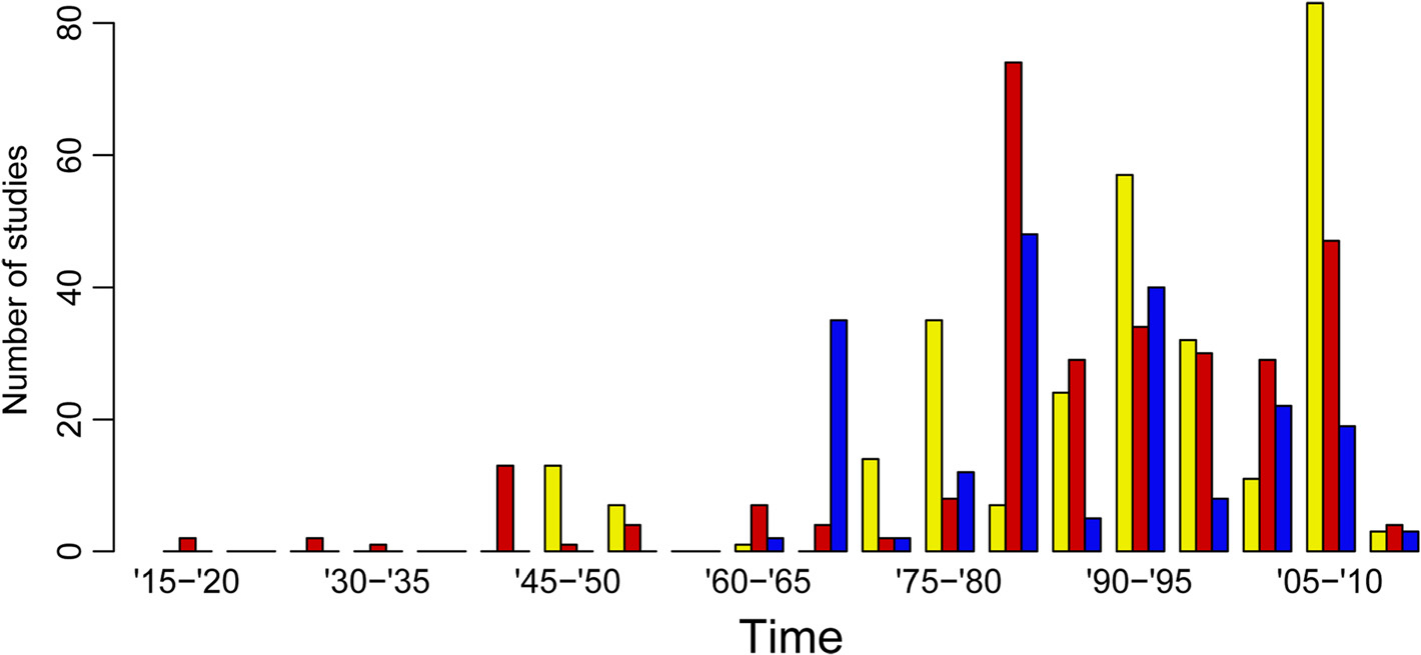
**Temporal distribution of MMRR experiments according to year of publication.** Yellow, red and blue bars for *Aedes*, *Anopheles* and *Culex*, respectively.

The frequency of MMRR records by year of reporting and genus is presented in Figure 4. MMRR studies on vector mosquitoes were first reported for anopheline mosquitoes a century ago, specifically those studying *An. albimanus* in the Panama Canal Zone [36]. MMRR studies investigating *Aedes* vectors were first reported in the 1940s [37] as were those for *Culex* mosquitoes [38]. It was not until the late 1970s, however, that MMRR studies began to be used more widely, with 87.8%, 87.6% and 80.1% of the MMRR data assembled for *Aedes*, *Anopheles* and *Culex* species, respectively, reported from 1975 onwards.

#### ***Disaggregated MMRR data***

**Figure 5:**
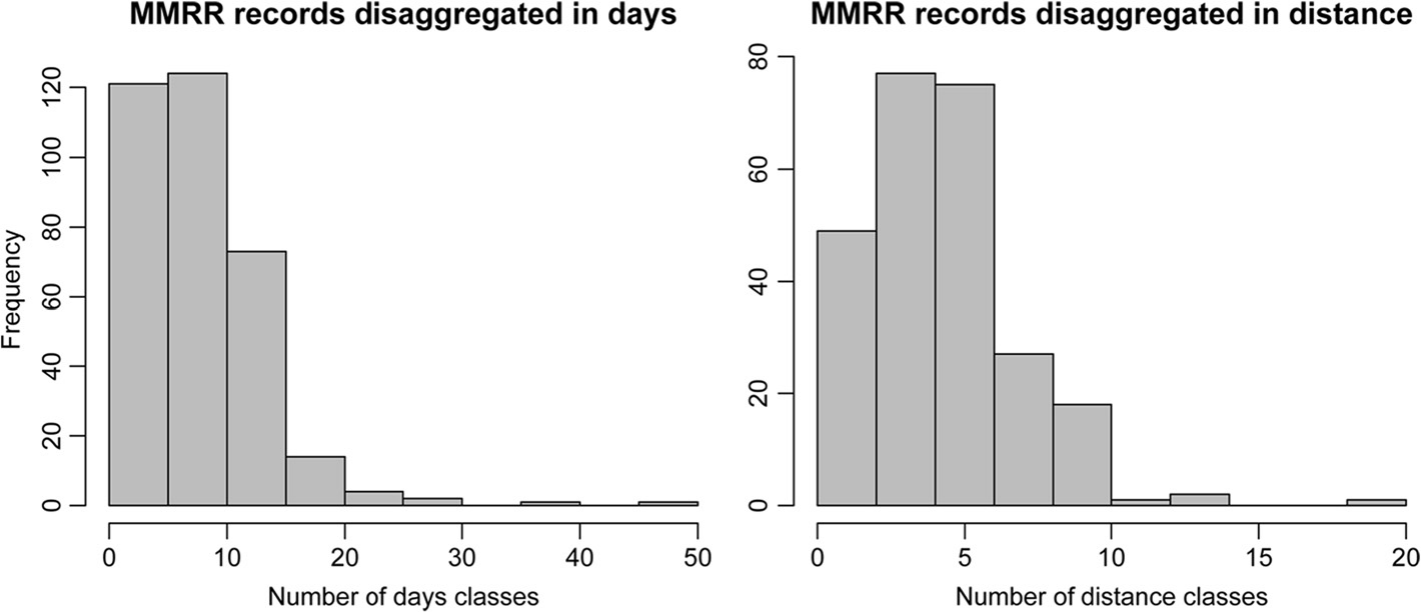
**Data disaggregation in time and distance as reported by MMRR studies.** X axes represent the number of classes into which data were disaggregated (i.e. number of days or distances for which recapture data were available).

There was variability in the way the data were aggregated for analysis and then reported, limiting the ability to disaggregate them back to the spatial and temporal resolution at which they were collected. Almost half of the MMRR data assembled (n = 355; 45.9%) were presented in a way that allowed disaggregating the information in time (i.e., daily recaptures; n = 340) and/or distance (i.e., recaptures by distance from the release point; n = 250). This proportion was similar for all three genera. Although the level of disaggregation (i.e., number of sub-records resulting from a single MMRR data record) varied considerably across studies, most reported recapture data for at least ten different days and/or five or more different distance classes (Figure 5). Twenty-seven percent of the studies presenting disaggregated data had them available by both day and distance. Disaggregated data were reported more commonly for *Ae. aegypti*, *An. culicifacies* and *Cx. tarsalis* (Table 3). Whenever available, disaggregated data also included the number of unmarked mosquitoes captured by day; this information, however, was reported less frequently.

### Methodologies used in MMRR studies

**Figure 6:**
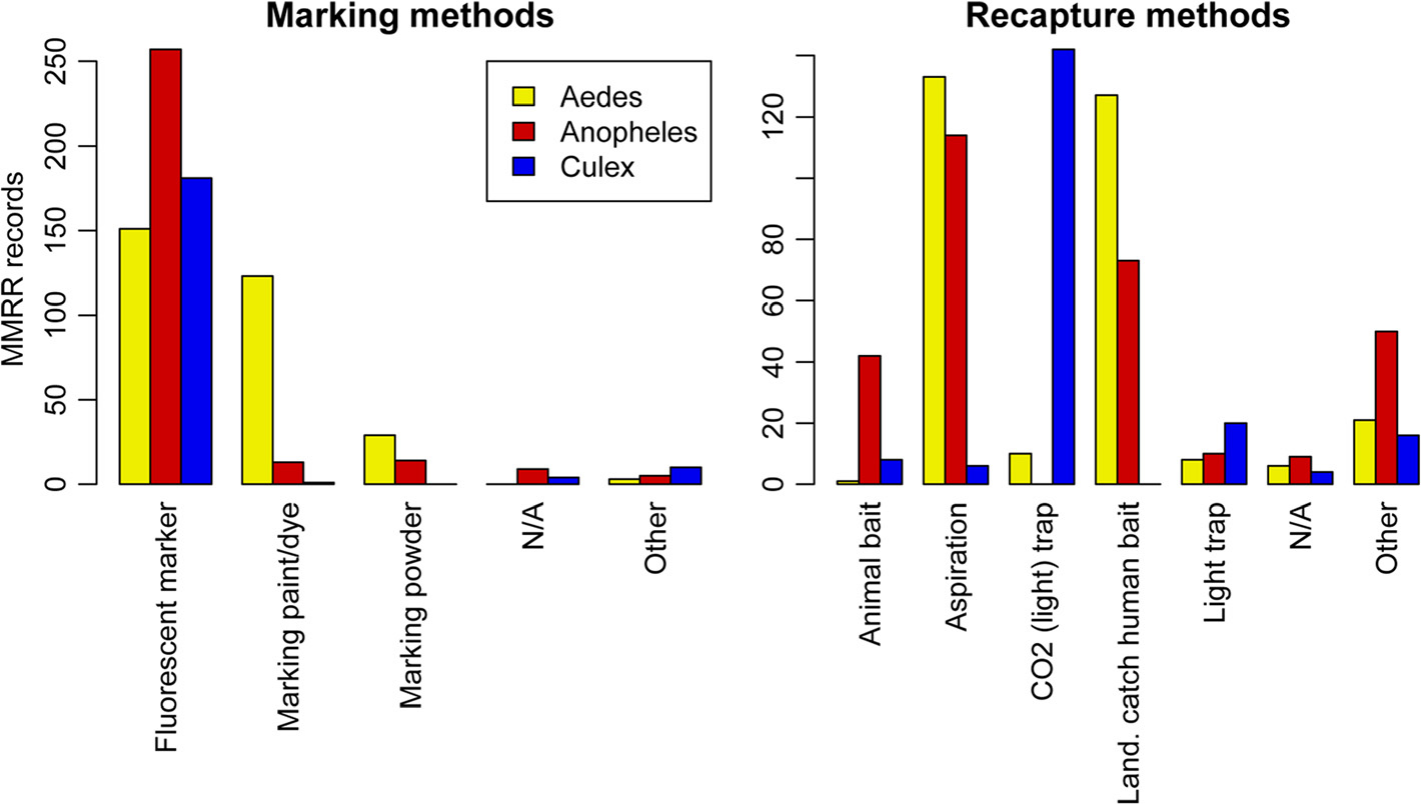
Frequency of mark and recapture methods by genus.

Different methods were used to source, mark, release and recapture adult female mosquitoes in MMRR studies (Table 4 and Figure 6).

**Table 4:** Frequency of key characteristics of release and recapture methods

**Variable**	**Value**^**¥**^	**All (n = 774)**	***Aedes *** **(n = 287)**	***Anopheles *** **(n = 291)**	***Culex *** **(n = 196)**
Origin of mosquitoes for release	Field-collected adults	52.6%	34.0%	73.7%	49.7%
	Field-collected immatures	23.4%	27.2%	9.7%	37.0%
	Lab-sourced adults	22.3%	38.9%	16.2%	7.4%
	Mixed	1.7%	0.0%	0.4%	5.8%
	N/S	0.0%	0.0%	0.0%	0.0%
Release events	Single	70.3%	73.2%	61.0%	78.8%
	Multiple	24.4%	25.3%	32.8%	11.6%
	N/S	5.3%	1.5%	6.2%	9.5%
Release sites	Single	69.6%	58.1%	73.7%	79.9%
	Multiple	30.3%	41.9%	25.9%	20.1%
	N/S	0.1%	0.0%	0.4%	0.0%
Release location	Indoors	27.6%	41.1%	34.0%	0.0%
	Outdoors	61.0%	53.2%	56.0%	78.8%
	Both	0.7%	0.0%	1.9%	0.0%
	N/S	10.7%	5.7%	8.1%	21.2%
Recapture location	Indoors	29.3%	47.9%	29.7%	2.6%
	Outdoors	45.2%	41.5%	27.4%	74.6%
	Both	17.1%	6.8%	38.6%	2.1%
	N/S	8.4%	3.8%	4.2%	20.6%
Age at release in days^†^	0	11.5%	12.0%	1.5%	17.9%
	1 to 2	19.2%	13.7%	32.4%	20.0%
	>2	35.5%	42.3%	42.6%	17.9%
	N/S	33.7%	32.0%	23.5%	44.2%
Feeding status at release	Blood-fed	18.7%	18.9%	31.7%	0.5%
	Sugar-fed	13.0%	11.7%	14.3%	13.2%

^¥^N/S = value not specified.

^†^Percentages are based on MMRR studies using field-collected immatures or lab-sourced adults (n = 338, n = 175, n = 68, n = 95 for all, *Aedes*, *Anopheles* and *Culex* studies, respectively).

#### ***Sourcing***

The origin of adult female mosquitoes used for marking was classified as follows: (i) wild adult mosquitoes caught and then marked were classified as “field-collected adults”; (ii) immature stages of mosquitoes caught and reared to adulthood prior to marking and releasing were classified as “field-collected immatures”; and (iii) adult female mosquitoes from established laboratory populations were classified as “lab-sourced adults”. About half (52.6%) of the MMRR experiments marked and released field-collected adult mosquitoes (Table 4). This varied considerably when looking at mosquito origin by genus. Most *Anopheles* experiments (73.7%) used field-collected adult females for release, whereas studies on *Aedes* sourced mosquitoes approximately equally (~1/3 each) from field-collected adults, field-collected immatures, and lab-sourced adults. Field-collected adult females and field-collected immature stages provided the main source for released adult *Culex* mosquitoes (86.7%; Table 4).

Two other important variables relevant to the release of adult female mosquitoes were age and feeding status at release. For the former, because the age of field-collected adult female mosquitoes is unknown and of mixed composition [33], only studies using field-collected immatures and lab-sourced adults were expected to report age at release, and were considered in these analyses (Table 4); however, 33.7% of such studies did not report this information. Most *Aedes* (42.3%) and *Anopheles* (42.6%) studies used mosquitoes that were more than two days old. Conversely, for *Culex* studies, nearly half did not report age (44.2%). In terms of feeding status at release, 18.7% of studies offered blood and 13% offered sugar solutions to marked mosquitoes prior to release. Notably, almost a third of anopheline MMRR studies offered adult females blood prior to release (31.7%), whereas virtually no *Culex* studies did (0.5%). If neither blood nor sugar feeding were explicitly reported, no feeding was assumed.

#### ***Marking***

A great variety of marking and recapturing methods was recorded in the database. For simplicity, these were grouped into the categories shown in Figure 6. Fluorescent markers were virtually the sole method used in MMRR experiments involving *Anopheles* and *Culex* mosquitoes (Figure 6). In *Aedes* studies, marking paints or dyes were also common.

#### ***Releasing***

The release of mosquitoes was categorized as either “single” or “multiple” in terms of both number of releases and number of release sites (Table 4). For example, a MMRR experiment may have released marked mosquitoes on a single date (releases = single), but from more than one site (release sites = multiple), and so forth. Single release events and sites predominated (70.3% and 69.6%, respectively; Table 4) and this was more evident for *Culex* MMRR studies, which used multiple release events and sites in only 11.6% and 20.1% of the cases, respectively. Multiple release sites were more common in studies involving *Aedes* (41.9%).

#### ***Recapturing***

For *Aedes* and *Anopheles* MMRR studies, aspiration (in various forms) and landing catches on human bait predominated. *Culex* studies mostly used CO_2_-baited traps with or without a light source (Figure 6).

The location of both release and recapture sites was classified as either “indoors” or “outdoors” (or a combination of “both”). With respect to release sites, most were outdoors (61%) and, in the case of *Culex* studies, no indoor releases were recorded, although more than a fifth of *Culex* MMRR studies did not specify the release location (Table 4). Recapture sites were more frequently located outdoors (45.2%) than indoors (29.3%) with 17.1% of studies recapturing both indoors and outdoors. The latter was more frequently the case in anopheline studies (38.6%), whereas in *Aedes* and *Culex* experiments combined recaptures (indoors and outdoors) were uncommon. As with release sites in *Culex* studies, recapture sites were predominantly located outdoors (74.6%) and very few reported indoor recaptures (2.6%), although a fifth of MMRR studies on *Culex* did not specify an indoor or outdoor location of the recapture sites (Table 4).

### Comparative analyses of recapture success

**Figure 7:**
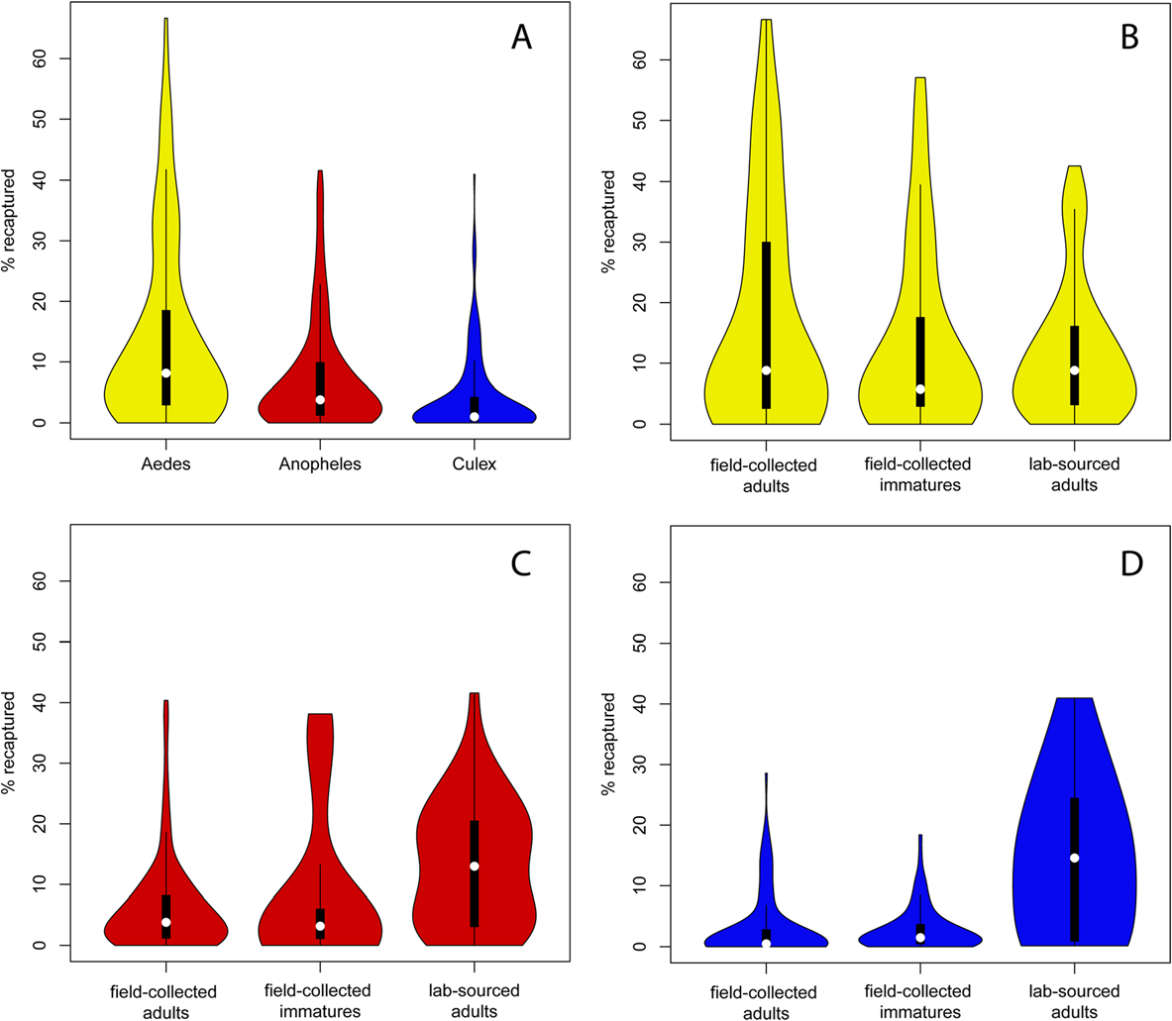
**Recapture success by (A) genus and source of mosquitoes for (B) *Aedes*, (C) *Anopheles*, (D) *Culex*.** White circles represent the medians, thick black lines the interquartile ranges and thin black lines the dispersion of the data. The violin-shaped contours represent kernel density plots.

**Figure 8:**
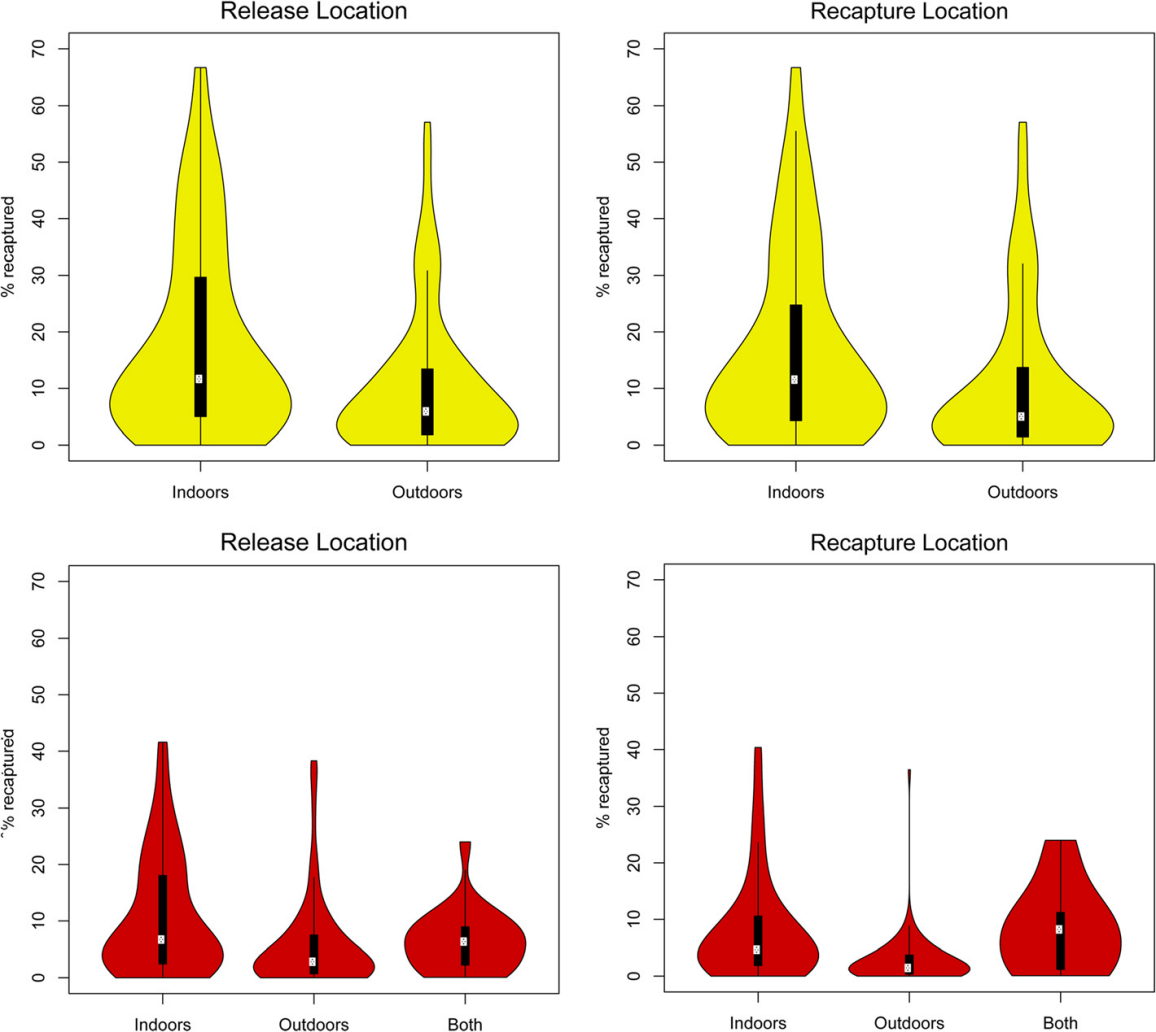
**Recapture success by location of release and recapture for *Aedes *(yellow) and *Anopheles *(red).***Culex* plots not shown given the predominance of outdoor release and recapture for this genus (Table 4). White circles represent the medians, thick black lines the interquartile ranges and thin black lines the dispersion of the data. The violin-shaped contours represent kernel density plots.

The median percent of adult female mosquitoes recaptured was significantly different amongst genera (Figure 7; Table 5). The *Culex* studies, in which mosquitoes were released and recaptured almost exclusively outdoors, showed the lowest recapture success (median = 1%) and the least variance, despite marking and releasing more individuals. The *Aedes* studies, by contrast, showed the highest recapture success (median = 8%) and the highest variance, despite marking and releasing the least numbers of mosquitoes. Median recapture effort, measured in the number of recapture days, was highest for *Aedes* (10 days) and lowest for *Culex* studies (5.5 days; Table 5). It is worth noting, however, that no correlation was found between recapture percent and recapture effort, measured either as number of recapture days or recapture stations. The influence of recapture and release location (indoors or outdoors) on recapture success was explored for *Aedes* and *Anopheles* (Figure 8). Indoor release and recapture resulted in higher recapture success for both genera.

**Table 5:** Medians and interquartile ranges (brackets) for adult female mosquitoes marked and released, percent recaptured and number of recapture days

**Genus**	**Number marked & released**	**% Recaptured**^**†**^	**Recapture days**
*Aedes* (n = 287)	208 (79–716)	8 (2–18)	10 (4.7-19.9)
*Anopheles* (n = 291)	678 (182–2428)	3 (1–9.5)	8 (5–11)
*Culex* (n = 196)	4067 (1559–12000)	1 (0.3-4)	6 (3–10)

^**†**^Kruskal-Wallis chi-squared = 117.3712, df = 2, p-value < 2.2e-16.

**Figure 9:**
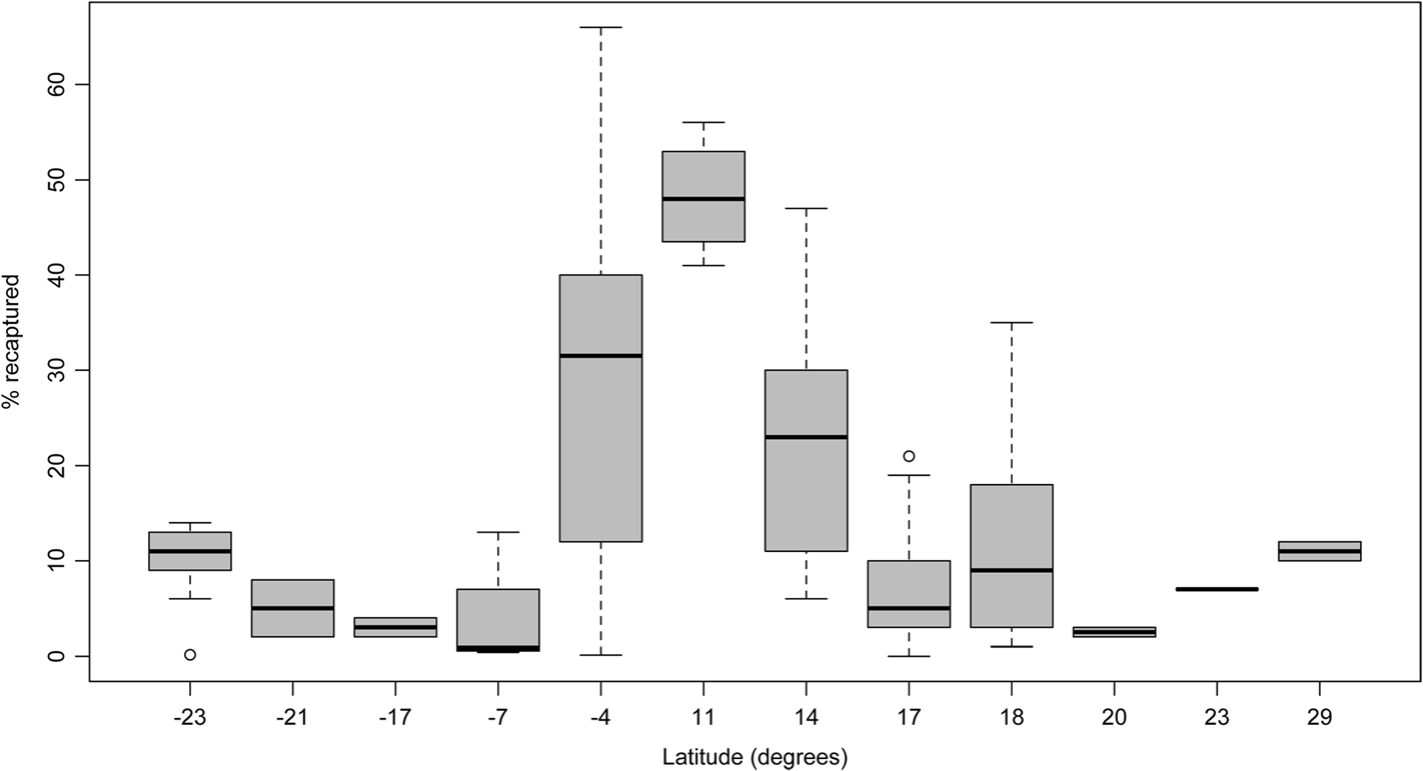
**Recapture success of *Ae. aegypti *adult female mosquitoes according to latitude.** The differences are highly statistically significant: logistic regression, Z = -70.64, p-value < 2*10^-16^.

Within-species variation of recapture success was explored for *Ae. aegypti,* which was the most commonly studied species across a wide range of habitats. The median percentage of adult females released and subsequently recaptured was 11% with an interquartile range between 4–23.8%, which was similar to the dispersion shown by all the *Aedes* data combined (Table 5). This result indicated strong within-species variation in recapture success that suggested that factors, other than species, strongly influence experimental outcomes. Figure 9 plots the recapture success of *Ae. aegypti* by the geographic location (latitude) of the study area. There is a clear trend towards higher recapture success around equatorial latitudes that implies an influence of climate on the outcome of MMRR experiments, although other factors, such as housing design and human behavior should be considered.

### Potential bias in estimates of dispersal

**Figure 10:**
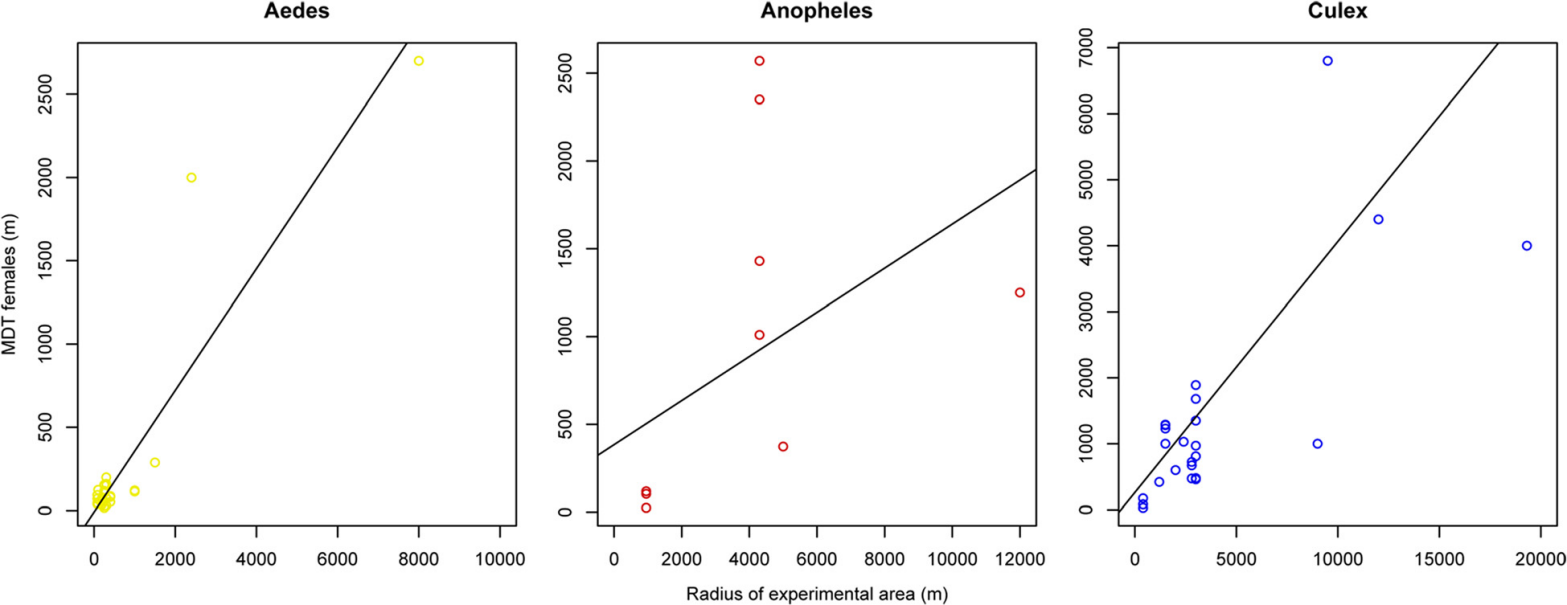
Estimated mean distance travelled (MDT) against the radius of the experimental area.

Figure 10 plots the estimated mean distance travelled (MDT) of adult female mosquitoes against the radius of the experimental area, given as the distance between the release location and the most distant recapture site. For all studies considered there was a clear positive correlation, suggesting that MDT estimates are strongly influenced by trap distance from the release point, and that study areas frequently were too small to document maximum flight range.

### Checklist

Cursory analysis of our database suggests a need to examine many aspects of MMRR studies in greater depth and to set standards for conducting and reporting studies for peer review. To begin that process, we developed a checklist for reporting MMRR data and a set of questions and key parameters that can be addressed and measured in future MMRR studies.

The checklist below is intended as a guide to a standard reporting system of MMRR experiments, ensuring that essential information is readily available from such studies.

#### ***Common background information***

• Where was the study conducted? (name of study area and geographical coordinates)

• When was the study conducted? (date of first release and date of last recapture and season corresponding to such period in the study area)

• What conditions characterized the area surrounding the study? (e.g. climatic variables such as wind speed, rainfall, temperature, relative humidity, and other data such as elevation and vegetative cover)

#### ***Marking, releasing and recapturing mosquitoes***

• What were the species and sex of mosquitoes marked and released?

• What was the source of mosquitoes marked and released? (“field-collected adults”, “field-collected immatures” or “lab-sourced adults”

• What type of marking method was used in the study?

• Were mosquitoes released from a single location or from multiple locations?

• Were mosquitoes released indoors or outdoors, or a combination of both?

• Were mosquitoes offered blood and/or sugar prior to release?

• If mosquitoes were lab-sourced adults or field-collected immatures, what was their age at release, in days?

• How many releases were done through the course of the study, in days?

• How many recaptures were done and at what frequency through the course of the study, in days?

• What type or types of recapture methods were used?

• What was the maximum distance between the release point(s) and the most distant recapture site/trap?

• What were the numbers of mosquitoes marked and released, and subsequently recaptured, by sex and by day and distance?

• How many recapture stations were used and what was their spatial configuration with respect to the release site? (e.g. symmetrical, asymmetrical, circular, quadrangular, etc.) – ideally, include a map of the release and recapture locations.

#### ***How could MMRR studies better inform mosquito-borne pathogen transmission?***

• Develop standards to estimate the precision of bionomic parameter estimates, including numbers of recaptured mosquitoes required to produce statistically robust estimates.

• Develop methods to estimate the accuracy of bionomic parameter estimates, perhaps by cross-validating these estimates against other methods; e.g., compare MMRR survivorship estimates with those calculated from parity dissections.

• Develop study designs to identify systematic differences in mosquito bionomic parameters across seasons, ecological settings, genotypes or bio-geographical zones.

• Examine the genetic and environmental causes of differences in recapture success.

• Examine the effect of particular physiographic factors on vector movement.

• Use MMRR data to simulate pathogen transmission networks and gene flow between vector and parasite populations.

• Infer patterns of spread of insecticide resistance genes and efficacy of ITNs following evidence of patterns of vector movement from MMRR studies.

• Use data from MMRR studies for modeling vector population dynamics to inform population genomics data and use these data to understand patterns of gene flow.

#### ***Parameters that could be measured during MMRR experiments***

• Dispersal

Distance travelled per time period

Direction of movement through various habitat matrices

Impact of wind speed and direction

• Probability of survival – decrease in recaptures over time

• Gonotrophic duration – dissect recaptures for egg maturation and parity

• Estimate survival from parity

• Population size and density

• Addition rate needed to maintain population size over time

## Discussion

MMRR experiments are a powerful tool for the study of different aspects of mosquito behavior and ecology, many of which are essential for understanding mosquito-borne pathogen transmission. The database assembled describes 774 MMRR experiments on *Aedes*, *Anopheles* and *Culex* species documenting the release of 3,341,116 adult female mosquitoes between 1913 and 2010, of which 90,524 (2.7%) were recaptured. Despite the considerable resources invested in creating this wealth of data, we found that the methods used were highly heterogeneous and the representation of species and ecological contexts was *ad hoc*; i.e. not intended to be adapted for purposes other than planned by the investigations. Further careful study of this database will be essential to identify detailed patterns that may be taxa specific, critique methodology, and guide design of future studies so that they can be better aggregated to more reliably inform public health. Preliminary biologically and epidemiologically relevant insights were, nevertheless, gained from a crude description of the assembled MMRR data and summary analyses.

Recapture success varied significantly across studies largely due to biological differences amongst mosquito genera/species. Typically, recapture success was significantly lower for highly vagile outdoor *Culex* species, particularly when compared with the generally less dispersive indoor *Aedes* species, reflecting important inherent behavioural dissimilarities and the propinquity of species-specific life resources such as blood meal hosts, resting sites and oviposition sites. Poor recapture success also may reflect a decrease in collection effort as a function of distance from the point of release and losses by emigration from the study area. Within-genus, and even within-species, variation in recapture success was also informative, particularly for *Aedes* species. There were two important considerations to account for these differences.

The first concerns the ecological context in which adult mosquitoes were released and recaptured. Local climate variables such as temperature affect daily mosquito survival [33,39], whereas excessive wind may limit dispersive flight activity [35]. Additionally, local weather conditions, land use and landscape features, such as natural or artificial barriers, can affect dispersal [40,41]. It is important, therefore, to consider site-specific MMRR data [42]. By using latitude as a crude proxy of climatic and ecological conditions of the study areas, we determined that recapture success varied significantly according to geographic location even when considering only studies of *Ae. aegypti* (Figure 9). For this species, MMRR experiments had significantly greater recapture success in study sites closer to the equator, suggesting a strong influence of climate, socioeconomic factors, mosquito population structure and other environmental factors. Public health applications of MMRR studies would benefit from designs that attempt to understand how bionomic patterns vary by ecological context.

Second, variation in experimental outcomes can be explained by the diversity of methodologies used in marking and recapturing mosquitoes [33], some of which clearly influenced recapture success. Different marking techniques, for example, can affect mosquito survivorship and behavior in different ways. If marking reduced longevity, this would limit the number of mosquitoes that were subsequently recaptured and affect estimates of dispersal and survival [33], even though common marking techniques applied correctly would not be expected to affect mosquito survival [43]. Similarly, laboratory sourced adult mosquitoes may exhibit atypical dispersal, activity rhythms and survival in MMRR experiments because of different behaviour, nutritional status or body size compared to their wild counterparts [33,44]. The distribution, density and frequency of operation of sampling assets critically influenced recapture success. Typically, the more recapture attempts that were made near the release site, the greater the recapture success. This approach could, however, compromise information on daily or net dispersal.

Although it is fair to conclude that the MMRR studies we examined have contributed to an improved, basic understanding of mosquito biology and behavior that are important for pathogen transmission, it remains unclear how effectively these studies can be used to inform transmission models. Our preliminary results highlight intriguing patterns, but how well those patterns can be generalized across species or different ecological contexts is still uncertain and will be addressed in subsequent work on the MMRR database. Aspirational goals for modeling pathogen transmission include development of functions that could assign baseline parameter values to mosquito species in different ecological contexts. An evidential basis for doing so would require understanding the ecological factors that account for variability in mosquito feeding habits, feeding frequency, survival, dispersal and population density. Our database provides a basis for examining existing methodologies and their potential to address these issues, but a cursory examination of the data suggests that the *ad hoc* approach will not provide the information required to develop ecological theory for medical entomology. Some thought must be given to how studies should be designed and where studies must be conducted to describe a mechanistic basis for phenomenological descriptions of mosquito behavior. Next steps will focus on critical questions about ideal methodologies and study designs. It is our hope that this assembly of data will motivate such future studies and increased efforts of standardization.

It should be noted that the database has a global scope and its development was based on comprehensive searches and relaxed inclusion criteria for MMRR studies. Despite an extensive effort, the database revealed a general paucity of MMRR data and limited spatial coverage. The MMRR data were available from only a handful of geographically clustered study sites across the world (Figures 1, 2 and 3), due, in part, to the specific objectives of associated research programs. Lack of spatial coverage is of obvious concern, particularly in light of the wide geographic distribution of mosquito vectors and the pathogens they transmit, including malaria, dengue, lymphatic filariasis, and some of the widely spread arboviral encephalitides [3,31,45,46]. For example, our database showed that even though *Ae. aegypti*, the primary vector of dengue viruses globally, has been investigated more than any other vector mosquito, MMRR data for this species originated from only 22 different study areas. Data for *An. gambiae* s.l. are even scarcer and are limited to eleven study areas scattered across six sub-Saharan African countries. This is a disappointing finding given that this species complex is responsible for most of the transmission and burden of *Plasmodium falciparum* malaria in the region [26]. Although our results suggest that many aspects of mosquito ecology that are important for transmission differ by ecological context, once the MMRR studies are broken down by geographical location and species, the amount of data available to examine geographic variation for the most important vectors of human infectious diseases remains limited.

For the past hundred years MMRR experiments have been used mainly to investigate adult mosquito dispersal and survivorship, two indisputably critical components of mosquito-borne pathogen transmission dynamics [42,47-49]. Adult mosquito dispersal can be estimated with a variety of MMRR approaches [33]. Investigators in most studies released adults at a central point and then recaptured them as they dispersed over time at traps placed in a variety of configurations and at different distances and sampling densities from the release point [50,51]. However, few studies adjusted recapture success by the reduction in sampling effort as a function of distance from the release point, which generally decreased as the square of the radius. The daily survival rate of adult individuals in the field, on the other hand, is commonly estimated through fitting single release and multiple recapture data to exponential models that assume age-independent survival rates [52], although the validity of this assumption has been questioned [42,53,54]. Our database was developed with the spatial resolution of MMRR data derived from dispersal experiments and the temporal resolution provided by survival experiments in mind. This means that despite the overall relative paucity of MMRR experiments, the database contains substantial amounts of raw, disaggregated release-recapture data that merit exploration for estimating dispersal and survival parameters, something that is of particular interest for mathematical and simulation models of mosquito-borne pathogen transmission.

## Conclusions

The aggregated MMRR database presented here contains a wealth of information describing mosquito populations, their movements and demography. Consequently, it constitutes the basis for critically analyzing one of the standard methods used by medical entomologists to measure the components of vectorial capacity, or the entomological potential for pathogen transmission. Importantly, the breadth of the assembled data can be used to reveal important phenomena and differences among taxa that are not apparent with any one MMRR study. The data assembled are directly applicable to an improved understanding of pathogen transmission by mosquitoes and the public health impact of vector control. More careful analyses that consider how different methods might affect the reported results remain to be done.

The database is available to anyone as *.csv files (Additional file 3). We hope that the methodologies used and results will be critiqued with the goal of stimulating improvements within the limitations of good ethical practices and biosafety, which ultimately constrain the design of MMRR studies, especially within areas supporting pathogen transmission. To that end, we developed a checklist with recommended guidelines for those who are designing new studies and who may want to augment this database.

## Abbreviations

MMRR: Mosquito mark-release-recapture; MDT: Mean distance travelled.

## Competing interests

The authors declare that they have no competing interests.

## Authors’ contributions

DLS, TWS and CAG designed the study. CAG assembled the MMRR database. CAG, RCR, TAP, DLS and TWS conceived the analyses and wrote the first draft of the paper. All authors helped in the interpretation of results, recommendations and read and approved the final draft of the paper.

## Supplementary Material

Additional file 1 **List and description of all relevant data fields in tables within Additional file **3. Click here for file

Additional file 2 List of references from which the MMRR data were extracted. Click here for file

Additional file 3 Compressed folder containing the raw MMRR data as three separate *.csv files. Click here for file
