# Supplementary material for: A global assembly of adult female mosquito mark-release-recapture data to inform the control of mosquito-borne pathogens
Source: Parasit Vectors. 2014 Jun 19;7:276. doi: 10.1186/1756-3305-7-276 (PMC4067626; doi:10.1186/1756-3305-7-276)
Supplement: Additional file 1 — List and description of all relevant data fields in tables within Additional file 3. [file 1756-3305-7-276-S1.pdf]

ADDITIONAL FILE 1

Table. List of data fields in the main database table (MMRRdata_main.csv within Additional file 3). Time and distance tables (MMRRdata_time.csv and MMRRdata_distance.csv, within Additional file 3) use the same nomenclature. For the former, the fields “day” and “day2” refer to the number of days passed after first release (when “day2” is available, a period of time was clearly defined). For the latter, the fields “distance1” and “distance2” refer to the distance, in meters, between the release point and the location of the traps (when “distance2” is available, a distance range was clearly defined).

Field name	Field definition
ENL_ID	Unique identifier linking to each original source
MRR_ID	Unique identified for each MMRR record
date_st	First day of experiment (date of first release)
date_en	Last day of experiment (last date of last recapture)
species1, 2, 3	Mosquito species studied (up to three species, 1 being the main one)
cohort	Cohort details, if defined
mos_origin	Origin of mosquitoes marked and released (see main text for definitions)
inoutrel	Place of release (Indoors or Outdoors)
inoutrec	Place of recapture (Indoors or Outdoors)
blood	Yes if female mosquitoes were blood-fed prior to release
sugar	Yes if mosquitoes were sugar-fed prior to release
age	Age at release, in days
mark_meth1	Main marking method used
mark_meth2	Alternative marking method used
release_meth	Were mosquitoes released at a Single place or in Multiple places
capture_meth1	Main capture method used
capture_meth2	Alternative capture method used
capture_meth3	Alternative capture method used
releases_n	Number of releases
recaptures_n	Number of recaptures
time1	Time from first release to first recapture (days)
time2	Time from first release to last recapture (days)
trap_range	Approximate radius of trapping area (m)
trap_range_shape	Symmetry of trapping area: symmetric or asymmetric
trap_number	Number of traps or number of collecting locations
release_sites_location	Location of release site(s): centric or eccentric
n_released_f	Number of marked female mosquitoes released
n_captured_f	Number of unmarked female mosquitoes captured
n_recaptured_f	Number of marked female mosquitoes recaptured
rratef	Estimated recapture rate for females (n_recaptured_f/n_released_f)
Pop1_f	Estimated female population size by Lincoln index
Pop2_f	Estimated female population size by alternative method
Pop3_f	Estimated female population size by alternative method
Pop4_f	Estimated female population size by alternative method
DPS1_f	Estimated daily probability of female survival by primary method
DPS2_f	Estimated daily probability of female survival by alternative method
MDT_f	Estimated mean distance travelled (females)
MnDT_f	Estimated minimum distance travelled (females)
MxDT_f	Estimated maximum distance travelled (females)
GC	Estimated duration of the gonotrophic cycle in days
disagg_d	Yes if the record is disaggredated in time (days – linked to MMRRdata_time.txt via MRR_ID)
