# Supplementary material for: A global assembly of adult female mosquito mark-release-recapture data to inform the control of mosquito-borne pathogens
Source: Parasit Vectors. 2014 Jun 19;7:276. doi: 10.1186/1756-3305-7-276 (PMC4067626; doi:10.1186/1756-3305-7-276)
Supplement: Additional file 2 — List of references from which the MMRR data were extracted. [file 1756-3305-7-276-S2.pdf]

ADDITIONAL FILE 2

List of the 161 original sources for MMRR data. Numbers to the left of each reference correspond to the unique identifier field (ENL_ID) in the main database table (MMRRdata_main.csv file within Additional file 3).

7 Gu W, Muller G, Schlein Y, Novak RJ, Beier JC: **Natural plant sugar sources of Anopheles mosquitoes strongly impact malaria transmission potential.** *PLoS One* 2011, **6:**e15996.

8 Bellini R, Albieri A, Balestrino F, Carrieri M, Porretta D, Urbanelli S, Calvitti M, Moretti R, Maini S: **Dispersal and survival of Aedes albopictus (Diptera: Culicidae) males in Italian urban areas and significance for sterile insect technique application.** *J Med Entomol* 2010, **47:**1082-1091.

13 Marini F, Caputo B, Pombi M, Tarsitani G, della Torre A: **Study of Aedes albopictus dispersal in Rome, Italy, using sticky traps in mark-release-recapture experiments.** *Med Vet Entomol* 2010, **24:**361-368.

15 Baber I, Keita M, Sogoba N, Konate M, Diallo M, Doumbia S, Traore SF, Ribeiro JM, Manoukis NC: **Population size and migration of Anopheles gambiae in the Bancoumana Region of Mali and their significance for efficient vector control.** *PLoS One* 2010, **5:**e10270.

19 Lacroix R, Delatte H, Hue T, Reiter P: **Dispersal and survival of male and female Aedes albopictus (Diptera: Culicidae) on Reunion Island.** *J Med Entomol* 2009, **46:**1117-1124.

25 Maciel-de-Freitas R, Lourenco-de-Oliveira R: **Presumed unconstrained dispersal of Aedes aegypti in the city of Rio de Janeiro, Brazil.** *Rev Saude Publica* 2009, **43:**8-12.

26 Maciel-de-Freitas R, Eiras AE, Lourenco-de-Oliveira R: **Calculating the survival rate and estimated population density of gravid Aedes aegypti (Diptera, Culicidae) in Rio de Janeiro, Brazil.** *Cad Saude Publica* 2008, **24:**2747-2754.

28 Tsuda Y, Komagata O, Kasai S, Hayashi T, Nihei N, Saito K, Mizutani M, Kunida M, Yoshida M, Kobayashi M: **A mark-release-recapture study on dispersal and flight distance of Culex pipiens pallens in an urban area of Japan.** *J Am Mosq Control Assoc* 2008, **24:**339-343.

31 Lapointe DA: **Dispersal of Culex quinquefasciatus (Diptera: Culicidae) in a Hawaiian rain forest.** *J Med Entomol* 2008, **45:**600-609.

33 Harrington LC, Francoisevermeylen, Jones JJ, Kitthawee S, Sithiprasasna R, Edman JD, Scott TW: **Age-dependent survival of the dengue vector Aedes aegypti (Diptera: Culicidae) demonstrated by simultaneous release-recapture of different age cohorts.** *J Med Entomol* 2008, **45:**307-313.

39 Midega JT, Mbogo CM, Mwnambi H, Wilson MD, Ojwang G, Mwangangi JM, Nzovu JG, Githure JI, Yan G, Beier JC: **Estimating dispersal and survival of Anopheles gambiae and Anopheles funestus along the Kenyan coast by using mark-release-recapture methods.** *J Med Entomol* 2007, **44:**923-929.

40 Achee NL, Grieco JP, Andre RG, Rejmankova E, Roberts DR: **A mark release-recapture study to define the flight behaviors of Anopheles vestitipennis and Anopheles albimanus in Belize, Central America.** *J Am Mosq Control Assoc* 2007, **23:**276-282.

48 Maciel-de-Freitas R, Codeco CT, Lourenco-de-Oliveira R: **Daily survival rates and dispersal of Aedes aegypti females in Rio de Janeiro, Brazil.** *Am J Trop Med Hyg* 2007, **76:**659-665.

57 Elizondo-Quiroga A, Flores-Suarez A, Elizondo-Quiroga D, Ponce-Garcia G, Blitvich BJ, Contreras-Cordero JF, Gonzalez-Rojas JI, Mercado-Hernandez R, Beaty BJ, Fernandez-Salas I: **Gonotrophic cycle and survivorship of Culex quinquefasciatus (Diptera: Culicidae) using sticky ovitraps in Monterrey, northeastern Mexico.** *J Am Mosq Control Assoc* 2006, **22:**10-14.

59 Achee NL, Grieco JP, Andre RG, Rejmankova E, Roberts DR: **A mark-release-recapture study using a novel portable hut design to define the flight behavior of Anopheles darlingi in Belize, Central America.** *J Am Mosq Control Assoc* 2005, **21:**366-379.

61 Yamar BA, Diallo D, Kebe CM, Dia I, Diallo M: **Aspects of bioecology of two Rift Valley Fever Virus vectors in Senegal (West Africa): Aedes vexans and Culex poicilipes (Diptera: Culicidae).** *J Med Entomol* 2005, **42:**739-750.

62 Russell RC, Webb CE, Williams CR, Ritchie SA: **Mark-release-recapture study to measure dispersal of the mosquito Aedes aegypti in Cairns, Queensland, Australia.** *Med Vet Entomol* 2005, **19:**451-457.

64 Fabian MM, Toma T, Tsuzuki A, Saita S, Miyagi I: **Mark-release-recapture experiments with Anopheles saperoi (Diptera: Culicidae) in the Yona Forest, northern Okinawa, Japan.** *Southeast Asian J Trop Med Public Health* 2005, **36:**54-63.

65 Harrington LC, Scott TW, Lerdthusnee K, Coleman RC, Costero A, Clark GG, Jones JJ, Kitthawee S, Kittayapong P, Sithiprasasna R, Edman JD: **Dispersal of the dengue vector Aedes aegypti within and between rural communities.** *Am J Trop Med Hyg* 2005, **72:**209-220.

67 dos Santos RL, Forattini OP, Burattini MN: **Anopheles albitarsis s.l. (Diptera: Culicidae) survivorship and density in a rice irrigation area of the state of Sao Paulo, Brazil.** *J Med Entomol* 2004, **41:**997-1000.

68 Watson TM, Saul A, Kay BH: **Aedes notoscriptus (Diptera: Culicidae) survival and dispersal estimated by mark-release-recapture in Brisbane, Queensland, Australia.** *J Med Entomol* 2000, **37:**380-384.

73 Reisen WK, Lothrop HD, Lothrop B: **Factors influencing the outcome of mark-release-recapture studies with Culex tarsalis (Diptera: Culicidae).** *J Med Entomol* 2003, **40:**820-829.

78 dos Santos RL, Forattini OP, Burattini MN: **Laboratory and field observations on duration of gonotrophic cycle of Anopheles albitarsis s.l. (Diptera: Culicidae) in southeastern Brazil.** *J Med Entomol* 2002, **39:**926-930.

80 Cho SH, Lee HW, Shin EH, Lee HI, Lee WG, Kim CH, Kim JT, Lee JS, Lee WJ, Jung GG, Kim TS: **A mark-release-recapture experiment with Anopheles sinensis in the northern part of Gyeonggi-do, Korea.** *Korean J Parasitol* 2002, **40:**139-148.

85 Harrington LC, Buonaccorsi JP, Edman JD, Costero A, Kittayapong P, Clark GG, Scott TW: **Analysis of survival of young and old Aedes aegypti (Diptera: Culicidac) from Puerto Rico and Thailand.** *J Med Entomol* 2001, **38:**537-547.

86 Tsuda Y, Takagi M, Wang S, Wang Z, Tang L: **Movement of Aedes aegypti (Diptera: Culicidae) released in a small isolated village on Hainan Island, China.** *J Med Entomol* 2001, **38:**93-98.

91 Tsuda Y, Takagi M, Suwonkerd W: **A mark-release-recapture study on the spatial distribution of host-seeking anophelines in northern Thailand.** *J Vector Ecol* 2000, **25:**16-22.

93 Tsuda Y, Takagi M, Toma T, Sugiyama A, Miyagi I: **Mark-release-recapture experiment with adult Anopheles minimus (Diptera: Culicidae) on Ishigaki Island, Ryukyu Archipelago, Japan.** *J Med Entomol* 1999, **36:**601-604.

94 Morrison AC, Costero A, Edman JD, Clark GG, Scott TW: **Increased fecundity of Aedes aegypti fed human blood before release in a mark-recapture study in Puerto Rico.** *J Am Mosq Control Assoc* 1999, **15:**98-104.

95 Muir LE, Kay BH: **Aedes aegypti survival and dispersal estimated by mark-release-recapture in northern Australia.** *Am J Trop Med Hyg* 1998, **58:**277-282.

96 Toure YT, Dolo G, Petrarca V, Traore SF, Bouare M, Dao A, Carnahan J, Taylor CE: **Mark-release-recapture experiments with Anopheles gambiae s.l. in Banambani Village, Mali, to determine population size and structure.** *Med Vet Entomol* 1998, **12:**74-83.

98 Quinones ML, Lines JD, Thomson MC, Jawara M, Morris J, Greenwood BM: **Anopheles gambiae gonotrophic cycle duration, biting and exiting behaviour unaffected by permethrin-impregnated bednets in The Gambia.** *Med Vet Entomol* 1997, **11:**71-78.

100 Costantini C, Li SG, Della Torre A, Sagnon N, Coluzzi M, Taylor CE: **Density, survival and dispersal of Anopheles gambiae complex mosquitoes in a west African Sudan savanna village.** *Med Vet Entomol* 1996, **10:**203-219.

102 Thomson MC, Connor SJ, Quinones ML, Jawara M, Todd J, Greenwood BM: **Movement of Anopheles gambiae s.l. malaria vectors between villages in The Gambia.** *Med Vet Entomol* 1995, **9:**413-419.

105 Mnzava AE, Rwegoshora RT, Wilkes TJ, Tanner M, Curtis CF: **Anopheles arabiensis and An. gambiae chromosomal inversion polymorphism, feeding and resting behaviour in relation to insecticide house-spraying in Tanzania.** *Med Vet Entomol* 1995, **9:**316-324.

107 Kramer VL, Carper ER, Beesley C, Reisen WK: **Mark-release-recapture studies with Aedes dorsalis (Diptera: Culicidae) in coastal northern California.** *J Med Entomol* 1995, **32:**375-380.

108 Trpis M, Hausermann W, Craig GB, Jr.: **Estimates of population size, dispersal, and longevity of domestic Aedes aegypti aegypti (Diptera: Culicidae) by mark-release-recapture in the village of Shauri Moyo in eastern Kenya.** *J Med Entomol* 1995, **32:**27-33.

109 Jensen T, Washino RK: **Comparison of recapture patterns of marked and released Aedes vexans and Ae. melanimon (Diptera: Culicidae) in the Sacramento Valley of California.** *J Med Entomol* 1994, **31:**607-610.

110 Fernandez-Salas I, Rodriguez MH, Roberts DR: **Gonotrophic cycle and survivorship of Anopheles pseudopunctipennis (Diptera: Culicidae) in the Tapachula foothills of southern Mexico.** *J Med Entomol* 1994, **31:**340-347.

115 Bryan JH, O'Donnell MS, Berry G, Carvan T: **Dispersal of adult female Culex annulirostris in Griffith, New South Wales, Australia: a further study.** *J Am Mosq Control Assoc* 1992, **8:**398-403.

117 Jaal Z, MacDonald WW: **A mark-release-recapture experiment with Anopheles lesteri paraliae in northwest Peninsular Malaysia.** *Ann Trop Med Parasitol* 1992, **86:**419-424.

119 O'Donnell MS, Berry G, Carvan T, Bryan JH: **Dispersal of adult females of Culex annulirostris in Griffith, New South Wales, Australia.** *J Am Mosq Control Assoc* 1992, **8:**159-165.

120 Rodriguez MH, Bown DN, Arredondo-Jimenez JI, Villarreal C, Loyola EG, Frederickson CE: **Gonotrophic cycle and survivorship of Anopheles albimanus (Diptera: Culicidae) in southern Mexico.** *J Med Entomol* 1992, **29:**395-399.

123 Chiang GL, Loong KP, Chan ST, Eng KL, Yap HH: **Capture-recapture studies with Anopheles maculatus Theobald (Diptera: Culicidae) the vector of malaria in peninsular Malaysia.** *Southeast Asian J Trop Med Public Health* 1991, **22:**643-647.

126 Reisen WK, Milby MM, Meyer RP, Pfuntner AR, Spoehel J, Hazelrigg JE, Webb JP, Jr.: **Mark-release-recapture studies with Culex mosquitoes (Diptera: Culicidae) in Southern California.** *J Med Entomol* 1991, **28:**357-371.

127 Jensen T, Washino RK: **An assessment of the biological capacity of a Sacramento Valley population of Aedes melanimon to vector arboviruses.** *Am J Trop Med Hyg* 1991, **44:**355-363.

128 Eldridge BF, Reeves WC: **Daily survivorship of adult Aedes communis in a high mountain environment in California.** *J Am Mosq Control Assoc* 1990, **6:**662-666.

133 Schreiber ET, Mulla MS, Chaney JD, Dhillon MS: **Dispersal of Culex quinquefasciatus from a dairy in southern California.** *J Am Mosq Control Assoc* 1988, **4:**300-304.

137 Trpis M, Hausermann W: **Dispersal and other population parameters of Aedes aegypti in an African village and their possible significance in epidemiology of vector-borne diseases.** *Am J Trop Med Hyg* 1986, **35:**1263-1279.

138 Hii JL: **Evidence for the existence of genetic variability in the tendency of Anopheles balabacensis to rest in houses and to bite man.** *Southeast Asian J Trop Med Public Health* 1985, **16:**173-182.

143 Rawlings P, Curtis CF: **Tests for the existence of genetic variability in the tendency of Anopheles culicifacies species B to rest in houses and to bite man.** *Bull World Health Organ* 1982, **60:**427-432.

144 Reisen WK, Mahmood F, Parveen T: **Anopheles culicifacies Giles: a release-recapture experiment with cohorts of known age with implications for malaria epidemiology and genetical control in Pakistan.** *Trans R Soc Trop Med Hyg* 1980, **74:**307-317.

146 Reisen WK, Aslam Y, Siddiqui TF, Khan AQ: **A mark-release-recapture experiment with Culex tritaeniorhynchus Giles.** *Trans R Soc Trop Med Hyg* 1978, **72:**167-177.

149 Macdonald WW, Sebastian A, Maung Tun M: **A mark-release-recapture experiment with Culex pipiens fatigans in the village of Okpo, Burma.** *Ann Trop Med Parasitol* 1968, **62:**200-209.

167 Tietze NS, Stephenson MF, Sidhom NT, Binding PL: **Mark-recapture of Culex erythrothorax in Santa Cruz County, California.** *J Am Mosq Control Assoc* 2003, **19:**134-138.

171 Walton WE, Workman PD, Tempelis CH: **Dispersal, survivorship, and host selection of Culex erythrothorax (Diptera: Culicidae) associated with a constructed wetland in southern California.** *J Med Entomol* 1999, **36:**30-40.

174 Pumpuni CB, Walker ED: **Population size and survivorship of adult Aedes triseriatus in a scrap tireyard in northern Indiana.** *J Am Mosq Control Assoc* 1989, **5:**166-172.

176 Charlwood JD, Bryan JH: **A mark-recapture experiment with the filariasis vector Anopheles punctulatus in Papua New Guinea.** *Ann Trop Med Parasitol* 1987, **81:**429-436.

953 Charlwood JD, Alecrim WA: **Capture-recapture studies with the South American malaria vector Anopheles darlingi, Root.** *Ann Trop Med Parasitol* 1989, **83:**569-576.

954 Birley MH, Charlwood JD: **The effect of moonlight and other factors on the oviposition cycle of malaria vectors in Madang, Papua New Guinea.** *Ann Trop Med Parasitol* 1989, **83:**415-422.

961 Charlwood JD, Graves PM: **The effect of permethrin-impregnated bednets on a population of Anopheles farauti in coastal Papua New Guinea.** *Med Vet Entomol* 1987, **1:**319-327.

1146 Ciota AT, Drummond CL, Drobnack J, Ruby MA, Kramer LD, Ebel GD: **Emergence of Culex pipiens from overwintering hibernacula.** *J Am Mosq Control Assoc* 2011, **27:**21-29.

1334 Ulloa A, Arredondo-Jimenez JI, Rodriguez MH, Fernandez-Salas I: **Mark-recapture studies of host selection by Anopheles (Anopheles) vestitipennis.** *J Am Mosq Control Assoc* 2002, **18:**32-35.

1336 McCall PJ, Mosha FW, Njunwa KJ, Sherlock K: **Evidence for memorized site-fidelity in Anopheles arabiensis.** *Trans R Soc Trop Med Hyg* 2001, **95:**587-590.

1365 Arredondo-Jimenez JI, Rodriguez MH, Washino RK: **Gonotrophic cycle and survivorship of Anopheles vestitipennis (Diptera: Culicidae) in two different ecological areas of southern Mexico.** *J Med Entomol* 1998, **35:**937-942.

1382 Hii JL, Birley MH, Sang VY: **Estimation of survival rate and oviposition interval of Anopheles balabacensis mosquitoes from mark-recapture experiments in Sabah, Malaysia.** *Med Vet Entomol* 1990, **4:**135-140.

1397 Bond JG, Rojas JC, Arredondo-Jimenez JI, Quiroz-Martinez H, Valle J, Williams T: **Population control of the malaria vector Anopheles pseudopunctipennis by habitat manipulation.** *Proc Biol Sci* 2004, **271:**2161-2169.

1429 Nelson RL, Milby MM, Grant CD: **Effects of fluorescent marker dusts on Culex tarsalis, a factor in mark-release-recapture studies.** In *Book Effects of fluorescent marker dusts on Culex tarsalis, a factor in mark-release-recapture studies* (Editor ed.^eds.). pp. 66,Äì68. City; 1980:66,Äì68.

1484 Renshaw M, others: **Host finding, feeding patterns and evidence for a memorized home range of the mosquito Aedes cantans.** *Medical and Veterinary Entomology* 1994, **8:**187-193.

1490 Milby MM, Reisen WK: **Estimation of vectorial capacity: vector survivorship.** *Bull Soc Vector Ecol* 1989, **14:**47,Äì54.

1497 Fouque F, Carinci R, Gaborit P, Issaly J, Bicout DJ, Sabatier P: **Aedes aegypti survival and dengue transmission patterns in French Guiana.** *Journal of Vector Ecology* 2006, **31:**390,Äì399.

1511 Maciel-De-Freitas R, Code√ßo CT, Louren√áO-De-Oliveira R: **Body size-associated survival and dispersal rates of Aedes aegypti in Rio de Janeiro.** *Medical and Veterinary Entomology* 2007, **21:**284-292.

1512 Reisen WK, Baker RH, Sakai RK, Mahmood F, Rathor HR, Raana K, Toqir G: **Anopheles culicifacies Giles: mating behavior and competitiveness in nature of chemosterilized males carrying a genetic sexing system.** *Annals of the Entomological Society of America* 1981, **74:**395,Äì401.

1513 Loong KP, Chiang GL, Eng KL, Chan ST, Yap HH: **Survival and feeding behaviour of Malaysian strain of Anopheles maculatus Theobald (Diptera: Culicidae) and their role in malaria transmission.** *Tropical Biomedicine* 1990, **7:**71-76.

1520 Nelson RL, Milby MM: **Dispersal and survival of field and laboratory strains of Culex tarsalis (Diptera: Culicidae).** *Journal of Medical Entomology* 1980, **17:**146-150.

1589 Reisen WK, Milby MM, Meyer RP: **Population dynamics of adult Culex mosquitoes (Diptera: Culicidae) along the Kern River, Kern County, California, in 1990.** *Journal of Medical Entomology* 1992, **29:**531-543.

1617 Maciel de Freitas R, Neto RB, Goncalves JM, Codeco CT, Lourenco-de-Oliveira R: **Movement of dengue vectors between the human modified environment and an urban forest in Rio de Janeiro.** *Journal of Medical Entomology* 2006, **43:**1112-1120.

1622 McDonald PT: **Population characteristics of domestic Aedes aegypti (Diptera: Culicidae) in villages on the Kenya Coast I. Adult survivorship and population size.** *Journal of Medical Entomology* 1977, **14:**42,Äì48.

1623 Curtis CF, Rawlings P: **A preliminary study of dispersal and survival of Anopheles culicifacies in relation to the possibility of inhibiting the spread of insecticide resistance.** *Ecological Entomology* 1980, **5:**11-17.

1640 Burkot TR, Graves PM, Paru R, Battistutta D, Barnes A, Saul A: **Variations in malaria transmission rates are not related to anopheline survivorship per feeding cycle.** *The American Journal of Tropical Medicine and Hygiene* 1990, **43:**321.

1724 Rawlings P, Davidson G: **The dispersal and survival of *Anopheles Culicifacies* Giles (Diptera: Culicidae) in a Sri Lankan village under malathion spraying.** *Bulletin of entomological research* 1982, **72:**139-144.

1727 Conway GR, Trpis M, McClelland GAH: **Population parameters of the mosquito Aedes aegypti (L.) estimated by mark-release-recapture in a suburban habitat in Tanzania.** *Journal of Animal Ecology* 1974, **43:**289,Äì304.

1737 Taylor C, Tour√© YT, Carnahan J, Norris DE, Dolo G, Traor√© SF, Edillo FE, Lanzaro GC: **Gene flow among populations of the malaria vector, Anopheles gambiae, in Mali, West Africa.** *Genetics* 2001, **157:**743-750.

1753 LaSalle MW, Dakin ME: **Dispersal of Culex salinarius in southwestern Louisiana.** *MOSQ NEWS* 1982, **42:**543-550.

1994 Reisen WK, Mahmood F, Parveen T: **Anopheles subpictus Grassi: observations on survivorship and population size using mark-release-recapture and dissection methods.** *Researches on Population Ecology* 1979, **21:**12-29.

2030 Sinsko MJ, Craig GB: **Dynamics of an isolated population of Aedes triseriatus (Diptera: Culicidae). I. Population size.** *Journal of Medical Entomology* 1979, **15:**89,Äì98.

2047 Lothrop H, Lothrop B, Palmer M, Wheeler S, Gutierrez A, Gomsi D, Reisen WK: **Evaluation of pyrethrin and permethrin ground ultra-low volume applications for adult Culex control in rural and urban environments of the Coachella Valley of California.** *Journal of the American Mosquito Control Association* 2007, **23:**190-207.

2064 Weidhaas DE, Breeland SG, Lofgren CS, Dame DA, Kaiser R: **Release of chemosterilized males for the control of Anopheles albimanus in El Salvador: IV. Dynamics of the test population.** *The American Journal of Tropical Medicine and Hygiene* 1974, **23:**298.

2073 McDonald PT: **POPULATION CHARACTERISTICS OF DOMESTIC AEDES AEGYPTI (DIPTERA: GULICIDAE) IN VILLAGES ON THE KENYA COAST II. Dispersal within and between villages.** *Journal of Medical Entomology* 1977, **14:**49,Äì53.

2110 Nutsathapana S, Sawasdiwongphorn P, Chitprarop U, Cullen JR, Gass RF, Green CA: **A mark-release-recapture demonstration of host-preference heterogeneity in *Anopheles minimus* Theobald (Diptera: Culicidae) in a Thai village.** *Bulletin of entomological research* 1986, **76:**313-320.

2135 Nelson RL, Milby MM, Reeves WC, Fine PEM: **Estimates of survival, population size, and emergence of Culex tarsalis at an isolated site.** *Annals of the Entomological Society of America* 1978, **71:**801,Äì808.

2214 Reisen WK, Mahmood F, Azra K: ***Anopheles culicifacies* Giles: Adult ecological parameters measured in rural Punjab province, Pakistan using capture-mark-release-**

recapture and dissection methods, with comparative observations on *An. stephensi* Liston and *An. subpictus* Grassi. *Researches on Population Ecology* 1981, **23:**39-60.

2268 Reisen WK, Lothrop HD: **Population ecology and dispersal of Culex tarsalis (Diptera: Culicidae) in the Coachella Valley of California.** *Journal of Medical Entomology* 1995, **32:**490-502.

2279 Reisen WK, Milby MM, Reeves WC, Meyer RP, Bock ME: **Population ecology of Culex tarsalis (Diptera: Culicidae) in a foothill environment of Kern County, California: temporal changes in female relative abundance, reproductive status, and survivorship.** *Annals of the Entomological Society of America* 1983, **76:**800,Äì808.

2840 Charlwood JD, Vij R, Billingsley PF: **Dry season refugia of malaria-transmitting mosquitoes in a dry savannah zone of east Africa.** *The American Journal of Tropical Medicine and Hygiene* 2000, **62:**726.

3565 McClelland GAH, McKenna RJ, Jolly DJ, Cahill TA: **Results of preliminary mark-release-recapture trials with Aedes nigromaculis.** *Proc Calif Mosq Control Assoc* 1973, **41:**107-108.

3656 Rawlings P, Curtis CF, Wickramasinghe MB, Lines J: **The influence of age and season on dispersal and recapture of Anopheles culicifacies in Sri Lanka.** *Ecological Entomology* 1981, **6:**307-319.

3677 Sempala SDK: **The Ecology of Aedes (Stegomyia) africanus (Theobald) in a Tropical Forest in Uganda: Mark Release Recapture Studies on a Female Adult Population.** *Insect Science and Its Application* 1981, **1:**211-224.

3723 Reisen WK, Mahmood F: **Relative abundance, removal sampling, and mark-release-recapture estimates of population size of *Anopheles culicifacies* and *An. stephensi* at diurnal resting sites in rural Punjab province, Pakistan.** *Mosquito news* 1981, **41:**22.

3985 Milby MM, Reisen WK, Meyer RP: **Mark-release-recapture studies with Culex mosquitoes along the Kern River, 1990.** In *Book Mark-release-recapture studies with Culex mosquitoes along the Kern River, 1990* (Editor ed.^eds.), vol. 59. pp. 58-61. City; 1991:58-61.

4794 Reisen WK, Mahmood F, Parveen T: **Seasonal trends in population size and survivorship of Anopheles culicifacies, An. stephensi and An. subpictus (Diptera: Culicidae) in rural Punjab Province, Pakistan.** *Journal of Medical Entomology* 1982, **19:**86-97.

4889 dos Santos RLC, Forattini OP: **Marcacao-soltura-recaptura para determinar o tamanho da populacao natural de Anopheles albitarsis ls (Diptera: Culicidae).** *Rev Saude Publica* 1999, **33:**309-313.

6056 Bogojevic MS, Hengl T, Merdic E: **Spatiotemporal monitoring of floodwater mosquito dispersal in Osijek, Croatia.** *Journal of the American Mosquito Control Association* 2007, **23:**99-108.

6075 Takagi M, Tsuda Y, Suzuki A, Wada Y: **Movement of individually marked Aedes albopictus females in Nagasaki, Japan.** *Trop Med* 1995, **37:**79-85.

6174 Buei K, Ito S, Nakamura H, Yoshida M: **Field studies on the gonotrophic cycle of Culex tritaeniorhynchus.** *Japanese Journal of Sanitary Zoology* 1980, **31:**57,Äì62.

6297 Ameneshewa B, others: **Blood-feeding behaviour of Anopheles arabiensis Patton (Diptera: Culicidae) in central Ethiopia.** *Journal of African Zoology* 1997, **111:**235-245.

6670 Bryan JH, Foley DH, Geary M, Carven CTJ: **Anopheles annulipes Walker (Diptera: Culicidae) at Griffith, New South Wales. 3. Dispersal of two sibling species.** *Australian Journal of Entomology* 1991, **30:**119-121.

6825 Haramis LD, Foster WA: **Survival and population density of Aedes triseriatus (Diptera: Culicidae) in a woodlot in central Ohio, USA.** *Journal of Medical Entomology* 1983, **20:**391-398.
6841 Laporta GZ: **Ecology of Culex quinquefasciatus and Culex nigripalpus at the Parque Ecológico do Tietê, São Paulo, Brasil.** 2007.
6897 Eyles DE, Bishop LK: **An experiment on the range of dispersion of Anopheles quadrimaculatus.** *American journal of epidemiology* 1943, **37:**239-245.
6900 Ordonez-Gonzalez JG, Mercado-Hernandez R, Flores-Suarez AE, Fernandez-Salas I: **The use of sticky ovitraps to estimate dispersal of Aedes aegypti in Northeastern Mexico.** *Journal of the American Mosquito Control Association* 2001, **17:**93-97.
6956 Walker ED, Copeland RS, Paulson SL, Munstermann LE: **Adult survivorship, population density, and body size in sympatric populations of Aedes triseriatus and Aedes hendersoni (Diptera: Culicidae).** *Journal of Medical Entomology* 1987, **24:**485-493.
7000 Pant CP, Yasuno M: **Field studies on the gonotrophic cycle of Aedes aegypti in Bangkok, Thailand.** *Journal of Medical Entomology* 1973, **10:**219-223.
7088 Weathersbee AA, Meisch MV: **Dispersal of Anopheles quadrimaculatus (Diptera: Culicidae) in Arkansas ricelands.** *Environmental Entomology* 1990, **19:**961-965.
7165 Bonnet DD, Worcester DJ: **The dispersal of Aedes albopictus in the territory of Hawaii.** *The American Journal of Tropical Medicine and Hygiene* 1946, **1:**465.
7172 Charlwood JD, Graves PM, Marshall TF: **Evidence for a 'memorized' home range in *Anopheles farauti* females from Papua New Guinea.** *Medical and Veterinary Entomology* 1988, **2:**101-108.
7426 Lounibos LP, Couto L, others: **Prompt mating of released Anopheles darlingi in western Amazonian Brazil.** *Journal of the American Mosquito Control Association* 1998, **14:**210-213.
8798 Roberts DR, Alecrim WD, Tavares AM, McNeill KM: **Field observations on the gonotrophic cycle of Anopheles darlingi (Diptera: Culicidae).** *Journal of Medical Entomology* 1983, **20:**189-192.
10060 Reisen WK, Sakai RK, Baker RH, Azra K: **ANOPHELES CULICIFACIES: OBSERVATIONS ON POPULATION ECOLOGY AND REPRODUCTIVE BEHAVIOR.** *Mosquito News* 1982, **42:**93.
10428 Dow RP, Reeves WC, Bellamy RE: **Dispersal of female Culex tarsalis into a larvicided area.** *The American Journal of Tropical Medicine and Hygiene* 1965, **14:**656-670.
10436 Self LS, Tun MM, Mathis HL, Abdulcader MH, Sebastian A: **Studies on infiltration of marked Culex pipiens fatigans into sprayed areas in Rangoon, Burma.** *Bulletin of the World Health Organization* 1971, **45:**379.
10465 Nayar JK, Provost MW, Hansen CW: **QUANTITATIVE BIONOMICS OF CULEX NIGRIPALPUS (DIPTERA: CULICIDAE) POPULATIONS IN FLORIDA 2. Distribution, dispersal and survival patterns.** *Journal of Medical Entomology* 1980, **17:**40,Äì50.
10478 Mahmood F, Reisen WK: **Duration of the gonotrophic cycles of Anopheles culicifacies Giles and Anopheles stephensi Liston, with observations on reproductive activity and survivorship during winter in Punjab Province, Pakistan.** *Mosq News* 1981, **41:**41-50.
10539 Carnevale P, Bosseno MF, Molinier M, Lancien J, Le Pont F, Zoulani A: **Etude du cycle gonotrophique d'Anopheles gambiae (Diptera, Culicidae) (Giles, 1902) en zone de foret degradee d'Afrique Centrale.** *Cahiers ORSTOM, Serie Entomologie medicale et Parasitologie* 1979, **17:**55-75.
10577 Correa RR, Lima FO, Coda D: **Observations on the Flight and Longevity in Nature of Anopheles albitarsis domesticus.** *Journal of the National Malaria Society* 1950, **9:**280-284.

10580 Dow RP: **The dispersal of Culex nigripalpus marked with high concentrations of radiophosphorus.** *Journal of Medical Entomology* 1971, **8:**353,Äì363.
10593 Eyles DE, Sabrosky CW, Russell JC: **Long-range dispersal of Anopheles quadrimaculatus.** In *Book Long-range dispersal of Anopheles quadrimaculatus* (Editor ed.^eds.), vol. 60. pp. 1265-1293. City: US Govt. Print. Office; 1945:1265-1293.
10596 Reisen WK, Yoshimura G, Reeves WC, Milby MM, Meyer RP: **The impact of aerial applications of ultra-low volume adulticides on Culex tarsalis populations (Diptera: Culicidae) in Kern County, California, USA, 1982.** *Journal of Medical Entomology* 1984, **21:**573-585.
10836 Charlwood JD, Graves PM, Birley MH: **Capture-recapture studies with mosquitoes of the group of Anopheles punctulatus Donitz (Diptera: Culicidae) from Papua New Guinea.** *Bull Entomol Res* 1986, **76:**211-227.
11149 Causey OR, Kumm HW: **Dispersion of forest mosquitoes in Brazil: Preliminary studies.** *The American Journal of Tropical Medicine and Hygiene* 1948, **1:**469.
11219 Gillies MT, Wilkes TJ: **A study of the age-composition of populations of Anopheles gambiae Giles and A. funestus Giles in North-Eastern Tanzania.** *Bulletin of Entomological Research* 1965, **56:**237,Äì262.
11227 Hitchcock JG: **Age composition of a natural population of Anopheles quadrimaculatus Say (Diptera: Culicidae) in Maryland, USA.** *Journal of Medical Entomology* 1968, **5:**125-134.
11283 Russell PF, Knipe FW, Rao TR, Putnam P: **Some experiments on flight range of Anopheles culicifacies.** *Journal of Experimental Zoology* 1944, **97:**135-163.
11346 Gillies MT: **Marking and release experiments with a tropical mosquito by the use of radioisotopes.** In *Book Marking and release experiments with a tropical mosquito by the use of radioisotopes* (Editor ed.^eds.). pp. 267. City; 1962:267.
11385 Kumm HW: **Studies in the Dispersion of Anopheles mosquitoes.** *The American Journal of Tropical Medicine and Hygiene* 1929, **1:**67.
11388 Causey OR, Kumm HW, Laemmert Jr HW: **Dispersion of forest mosquitoes in Brazil: further studies.** *The American Journal of Tropical Medicine and Hygiene* 1950, **1:**301.
11457 Smith GE, Watson RB, Crowell RL: **Observations on the flight range of Anopheles quadrimaculatus, Say.** *American journal of epidemiology* 1941, **34:**102-113.
13179 Trpis M, Hausermann W: **Demonstration of differential domesticity of Aedes aegypti (L.)(Diptera, Culicidae) in Africa by mark-release-recapture.** *Bulletin of Entomological Research* 1975, **65:**199,Äì208.
13213 Lutwama JJ, Mukwaya LG: **Mark-release-recapture studies on three anthropophilic populations of Aedes (Stegomyia) simpsoni complex (Diptera: Culicidae) in Uganda.** *Bulletin of Entomological Research* 1994, **84:**521-527.
13221 Wada Y, Kawai S, Oda T, Miyagi I, Suenaga O, Nishigaki J, Omori N, Takahashi K, Matsuo R, Itoh T, others: **Dispersal experiment of Culex tritaeniorhynchus in Nagasaki area (Preliminary report).** In *Book Dispersal experiment of Culex tritaeniorhynchus in Nagasaki area (Preliminary report)* (Editor ed.^eds.). City; 1969.
13309 Nelson RL, Milby MM: **Autogeny and blood-feeding by Culex tarsalis (Diptera: Culicidae) and the interval between oviposition and feeding.** *The Canadian Entomologist* 1982, **114:**515,Äì521.
13404 Takken W, Charlwood JD, Billingsley PF, Gort G: **Dispersal and survival of Anopheles funestus and A. gambiae sl (Diptera: Culicidae) during the rainy season in southeast Tanzania.** *Bulletin of Entomological Research* 1998, **88:**561-566.

13708 Ejercito A, Urbino CM: **Flight range of gravid and newly emerged Anopheles.** *Bulletin of the World Health Organization* 1951, **3:**663.
13748 Lines JD, Lyimo EO, Curtis CF: **Mixing of indoor-and outdoor-resting adults of Anopheles gambiae Giles sl and A. funestus Giles (Diptera: Culicidae) in coastal Tanzania.** *Bull Entomol Res* 1986, **76:**171-178.
13764 Gillies MT: **Studies on the dispersion and survival of Anopheles gambiae Giles in East Africa, by means of marking and release experiments.** *Bulletin of Entomological Research* 1961, **52:**99,Äì127.
14055 Abdel-Malek AA, Abdel-Aal MA: **Study of the Dispersion and Flight Range of Anopheles Sergenti Theo. in Siwa Oasis Using Radioactive Isotopes as Markers.** *Trans roy Soc trop Med Hyg* 1966, **35:**968-973.
14515 Valerio L, Facchinelli L, Ramsey JM, Scott TW: **Dispersal of Male Aedes aegypti in a Coastal Village in Southern Mexico.** *Am J Trop Med Hyg* 2012, **86:**665-676.
14518 Edman JD, Scott TW, Costero A, Morrison AC, Harrington LC, Clark GG: **Aedes aegypti (Diptera: Culicidae) movement influenced by availability of oviposition sites.** *J Med Entomol* 1998, **35:**578-583.
14519 Baker RH, Reisen WK, Sakai RK, Rathor HR, Raana K, Azra K, Niaz S: **Anopheles culicifacies: Mating behavior and competitiveness in nature of males carrying a complex chromosomal aberration.** *Annals of the Entomological Society of America* 1980, **73:**581-588.
14523 Geiger JC, Purdy WC, Tarbett RE: **Effective malaria control in a ricefield district with observations on experimental mosquito flights.** *Journal of the American Medical Association* 1919, **72:**844-847.
14530 Zetek J: **Behavior of *Anopheles albimanus* Wiede. and *tarsimaculata* Goeldi.** *Annals of the Entomological Society of America* 1915, **8:**221-271.
14531 Bailey SF, Eliason DA, Hoffmann BL: **Flight and dispersal of the mosquito *Culex tarsalis* Coquillett in the Sacramento Valley of California.** *HILGARDIA* 1965, **37:**73-113.
14554 Takagi M, Tsuda Y, Wada Y: **Movement and oviposition of released *Aedes albopictus* (Diptera: Culicidae) in Nagasaki, Japan.** *Jpn J Sanit Zool* 1995, **46:**131-138.
14561 Yasuno M, Rajagopalan PK, La Brecque GC: **Migration patterns of *Culex fatigans* around Delhi, India.** *Tropical Medicine* 1975, **17:**91-96.
14567 Eyles DE, Cox WW: **The measurement of a population of *Anopheles quadrimaculatus* Say.** *Journal of the National Malaria Society* 1943, **2:**71-83.
14570 Eddy GW, Roth AR, Plapp FW: **Studies on the flight habits of some marked insects.** *J Econ Entomol* 1962, **55:**603-607.
14571 Russell PF, Domingo S: **Flight range of the funestus-minimus subgroup of *Anopheles* in the Philippines.** *American Journal of Tropical Medicine* 1934, **14:**139-157.
14573 Reuben R, Yasuno M, Panicker KN, La Brecque GC: **The estimation of adult populations of *Aedes aegypti* at two localities in Delhi, India.** In *Book The estimation of adult populations of Aedes aegypti at two localities in Delhi, India* (Editor ed.^eds.). City: World Health Organization; 1972.
14574 Sharma VP: **Rate of insemination of female *Culex pipiens fatigans* Wied. moving from wells to the village.** In *Book Rate of insemination of female Culex pipiens fatigans Wied. moving from wells to the village* (Editor ed.^eds.). City: World Health Organization; 1975.
14595 Germain M, Herve JP, Geoffroy B: **Évaluation de la durée du cycle trophogonique d'*Aedes africanus* (Theobald), vecteur potentiel de fievre jaune, dans une galerie forestière du sud de la République Centr-africaine.** *Cahiers ORSTOM, Serie Entomologie Medicale et Parasitologie* 1974, **12:**127-133.

14598 Hervy JP: **Experience de marquage-lâcher-recapture portant sur *Aedes aegypti* Linné, en zone de savane soudanienne ouest-africaine. I. Le cycle trophogonique.** *Cahiers ORSTOM, Serie Entomologie Medicale et Parasitologie* 1977, **15:**353-364.
